# An R&D perspective on international trade and sustainable development

**DOI:** 10.1038/s41598-023-34982-3

**Published:** 2023-05-17

**Authors:** Lorenzo Costantini, Francesco Laio, Luca Ridolfi, Carla Sciarra

**Affiliations:** Politecnico di Torino, DIATI, 10129 Turin, Italy

**Keywords:** Environmental sciences, Environmental impact, Environmental social sciences, Environmental economics, Environmental impact, Sustainability, Statistics, Scientific data

## Abstract

Research and Development (R&D) is the common denominator of innovation and technological progress, supporting sustainable development and economic growth. In light of the availability of new datasets and innovative indicators, in this work, we introduce a novel perspective to analyse the international trade of goods through the lenses of the nexus R&D-industrial activities of countries. We propose two new indices, RDE and RDI, summarizing the R&D content of countries’ export and import baskets—respectively—and investigate their evolution in time, during the period 1995–2017, and space. We demonstrate the potential of these indices to shed new light on the evolution of R&D choices and trade, innovation, and development. In fact, compared to standard measures of countries’ development and economic growth (e.g., the Human Development Index among the others tested), these indices provide complementary information. In particular, tracing the trajectories of countries along the RDE-HDI plane, different dynamics appear for countries with increased HDI, which we speculate can be reasoned with countries’ availability of natural resources. Eventually, we identify two insightful applications of the indices to investigate further countries’ environmental performances as related to their role in international trade.

## Introduction

According to the endogenous growth theory^1^, innovation and technological progress are the main drivers of economic growth. These factors mainly rely on Research and Development^2,3^ (R&D), i.e., the systematic creative work aiming to increase the stock of knowledge and devoted to the creation and development of new products and procedures^4^. Typically, as countries become richer in their national income, their ability to invest in the R&D sector increases. These investments lead to the development of new products and/or more efficient production procedures, supporting job creation, industrial and economic growth^3,5–15^. In such a landscape, the trade of goods around the world plays a non-negligible role at the cross-section between economic growth and innovation. The trade of goods among countries almost doubled in monetary volume^16^ during the past fifteen years, with notable increment at the country level recorded in China, India, and low-wage economies^17,18^. The rise in countries’ commercial relationships is associated with economic well-being^19^ and innovation procedures^20,21^. Since the publication of the works by Hausmann et al.^22^ and Hidalgo et al.^23^, the concept that a country’s export basket composition is a crucial characteristic for determining its economic growth spreads out in the scientific community^24–29^. According to these studies^23–30^, a country can export a given product if it has the capability (i.e., the proper knowledge and know-how, such as infrastructures, human capital, and openness to entrepreneurship, among the others) required for the production of the good at hand. In this sense, economic growth is associated with the economic advantage a country gains by investing in the production and export of some given products rather than others^22^. The export outcomes of a country are considered to be those of its firms^23,26–28^, each one having the capabilities required to produce and export one or more products. These capabilities are supported and boosted by the social capital and health of institutions of the considered country^30,31^. Consequently, countries with a wide set of capabilities (gained by investing in new productive sectors) can diversify their export baskets, thus becoming central players in international trade through the supply of more knowledge-intensive goods^23,26^.

Against this background, our work provides a novel framework to describe international trade from the country-product perspective, considering the R&D content embedded in the exchanged goods. We compute the R&D content of nations’ trade baskets and study countries’ development dynamics between 1995 and 2017 from a trade-related R&D viewpoint. To detail the R&D content of the trade baskets of countries entails building a matching algorithm among different classifications of products and industrial activities, making use of existing standards and recent research contributions. Specifically, we capitalized on the work by Galindo-Rueda et al.^32^—who computed the R&D intensity associated with products classified according to their industrial sector—and made use of the trade data from the BACI-CEPII dataset^33^ to index the R&D content of countries’ trade baskets. We thus introduce two new indices, RDE and RDI, describing the R&D content in countries’ export and import baskets, respectively. Specifically, the RDE and RDI combine countries’ export and import baskets composition (in terms of products) with the R&D intensity of the exchanged goods. As we show, the countries’ R&D-trade dimension—herein identified through the RDE and RDI—relates to, but do not overlap with, the countries’ characteristics (such as Gross Domestic Product, Human Development Index, and Gross Expenditure in R&D), also detailing their development trajectories. These indices highlight non-trivial behaviours, especially among countries with low Gross Domestic Product per capita and Human Development Index. In light of these results, we speculate these indices provide information about countries’ development taking into account the composition and R&D content of countries’ trade baskets. Thus, our work aims to create further pillar support to bridge intersecting research works, also considering the ongoing thirst for the identification of complex indicators of development and benchmark of comparison. Furthermore, in light of the literature linking innovation, trade, and sustainable development^34–48^, we herein show two insightful uses of the indices related with environmental performances of countries. These applications detail the so-called *green products* and the CO_2_ emissions embedded in countries’ export. This analysis provides a novel perspective on the implications of investing in R&D at the country level and is a first step toward addressing the nexus among innovation, environmental performances, and green transition^37,39^.

## Results

### Assigning an R&D intensity value to traded products

Aiming to characterize international trade through an R&D perspective, we develop a two-step matching algorithm to build a bridge among products—as classified by the Harmonized System (HS) in use for the economic trade sector—and the R&D intensity by means of the industrial sector classification. The first step assigns each internationally traded product (identified by a 6-digit HS code^33^) to its industrial sector in the International Standard Industrial Classification (ISIC), making use of the conversion key provided by the Organisation for Economic Co-operation and Development (OECD, see Data and methods). The second step of such matching algorithm relies on the work by Galindo-Rueda et al.^32^, wherein the authors identified an R&D intensity value for all industrial sectors. The R&D intensity value of an industrial sector is the ratio between the R&D expenditure (at the country level) and the Gross Value Added for that given industry (notice that the ratio builds upon aggregated data for 29 countries in 2011, see Methods). To the best of our knowledge, such categorisation of the R&D intensity is the only existing one, and we assume its validity for all countries in the world despite being built through the data of a small subset of nations. Notice that inherited limitations pervade our work, which we discuss in the concluding part of the work. In fact, improving such classification would require further work on data collection at the national and international level, which exits the aim of the present work and towards which future data collection efforts should be addressed. (However, a more extensive discussion about the sensitivity of the input data, and its repercussion on our developed classification is present in the Supplemental Material).

**Figure 1:**
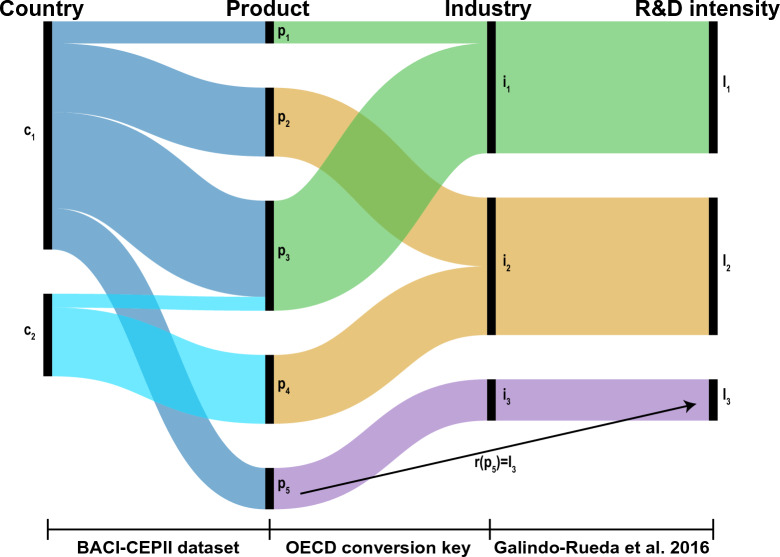
Qualitative description of the matching algorithm linking products to industries (step 1, central column) and industries to the corresponding R&D intensity (step 2, right column), also representing countries’ export or import baskets (left column). Using different colours (green, yellow, and purple) stresses the association of the products in their categorisation at the industrial level and in their R&D intensity. The size of each figure element (such as countries and products) and the width of the fluxes are proportional to the traded dollars. The black arrow describes the component of the vector **r** for the product $p_5$. The figure is produced using RAWGraphs (https://app.rawgraphs.io/).

In this work, the R&D intensity of a specific product is obtained via its categorisation in the industrial sector. We define **r** as the vector whose components are the R&D intensities associated with each product in the HS classification (in this work, 5002 goods are considered). Table 1 reports the basic statistical descriptors of the vector **r**, and Figure S1 of the Supplemental Material (SM) details the number of products as a function of the R&D intensity, showing that most of the goods have an R&D intensity smaller than 0.10. Once the vector **r** has been reconstructed, the R&D characterization of the countries’ trade basket becomes straightforward from the flow of traded dollars. Figure 1 eases the understanding of the matching algorithm, plotting its two steps and highlighting the creation of the vector **r**, which allows one to assign to each 6-digit code of HS products, an R&D intensity value. The figure also adds visual information on how the countries’ export and import baskets can be associated with a level of R&D intensity using its in- and out-flows of money. In the following subsections, we present the R&D description of international trade by analyzing the monetary flows at the world level (as retrieved from the BACI-CEPII dataset^33^) followed by an environment-oriented application of the R&D concepts.

**Table 1:** Statistical description of the vector **r**.

Quantity	Value
Minimum	0.0027
Maximum	0.3169
Median	0.0207
Mean	0.0516
Standard deviation	0.0636

### An R&D description of the global trade

Given the vector **r**, we rank its components in ascending order, aggregating the products into five classes of R&D intensity (Low, Medium-Low, Medium, Medium-High, and High R&D, see Data and methods) following Galindo-Rueda et al.^32^. To perform this aggregation, we create the matrix **M**, whose entries (*M*(*c*, *cl*)) represent how much a country *c* exports (or equivalently, imports) of a product *p* within the R&D class *cl* compared to the total monetary value of the global trade at a fixed year. Therefore, the value *M*(*c*, *cl*) is the market share (expressed as a percentage) country *c* holds in the R&D class *cl*. In mathematical terms:

1 $$\begin{aligned} M(c,cl)=\frac{\sum _{p\in cl} D(c,p)}{\sum _c \sum _p D(c,p)} \cdot 100 \end{aligned}$$

where **D** is the matrix reporting in each cell the dollars country *c* exports (imports) in product *p*. Both matrices **M** and **D** are adequately sub-indexed with the scripts “exp” or “imp” whether the calculation is computed for the export or import basket, respectively.

**Figure 2:**
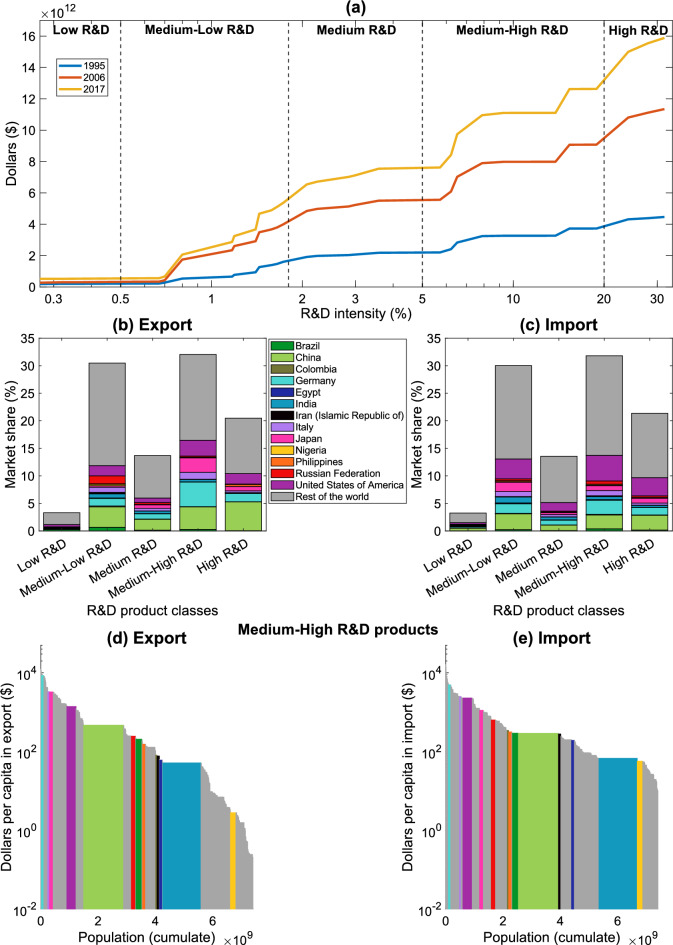
Description of the international trade in terms of products’ R&D intensity. Panel (**a**) reports the total volume of dollars traded as a function of the products’ R&D intensity in 1995, 2006, and 2017. Here, the black dashed lines delimit the R&D product classes^32^. Middle panels show the shares of global trade held by countries for each R&D intensity class (see Eq. 1) both in export (panel **b**) and import (panel **c**) in 2017. The bottom panels report the per-capita dollars traded by all countries (sorted from the largest to the smallest) for the products in the Medium-High R&D class in 2017 as a function of the population. We highlighted thirteen countries: Brazil, China, Colombia, Germany, Egypt, India, Iran, Italy, Japan, Nigeria, the Philippines, the Russian Federation, and the United States of America (which account for 46% of the total volume of the exported dollars in 2017); all the other countries are included in “Rest of the World”. The Figure is produced with MATLAB R2020b.

Figure 2 describes international trade from an R&D perspective, also considering its evolution in time. Figure 2a shows the dollars traded (at Purchasing Power Parity) as a function of the products’ R&D intensity for three out of 23 years of analysis, namely the first, middle, and last year (1995, 2006, and 2017). In general, the traded volume of dollars increased in time globally by 256% from 1995 to 2017, however the contribution of each R&D class to the total money flow remained almost constant in this period (please see Figure S2 of the SM). The highest rise in monetary fluxes is in the High R&D class (341% increase of money traded in 2017 compare to the 1995 volume), followed by the Medium-Low (247%) and Medium-High (229%) R&D products. Figures 2b and 2c report the countries’ market shares in each R&D class both in export and import in 2017, thus showing the elements of ${{\textbf {M}}}_{{{\textbf {exp}}}}$ and ${{\textbf {M}}}_{{{\textbf {imp}}}}$, respectively (refer to Eq. 1). The Medium-High R&D products form the largest share of world trade in 2017, followed by the Medium-Low, High, Medium, and Low R&D classes. The export and import histograms highlight differences in the countries’ (and regions’, see Fig. S3 of SM) behaviour. We comment on these results focusing mainly on thirteen countries (Brazil, China, Colombia, Germany, Egypt, India, Iran, Italy, Japan, Nigeria, the Philippines, Russian Federation, and the United States of America) that are representative of different geographical areas^49^ and R&D intensity embedded in their trade baskets. Countries in the Organisation for Economic Co-operation and Development (OECD) present notable market shares in export and import in all R&D classes, i.e., exhibiting large values in their corresponding elements in ${{\textbf {M}}}_{{{\textbf {exp}}}}$ and ${{\textbf {M}}}_{{{\textbf {imp}}}}$, respectively. Interestingly, these nations primarily export Medium-High R&D products and import goods in the Medium-Low R&D class (please see Figure S3 of SM). This behaviour preeminently characterizes Germany, Italy, Japan and the United States of America (USA), among those shown here. As Figs. 2 and S3 show, in 2017, East and South Asian countries held the second largest market shares in all R&D classes. Comparing panels 2b and 2c with the top panels of Figure S3, it is clear that China leads the trade panorama of this region, being a net-exporter—i.e., $M_{exp}(China,cl)>M_{imp}(China,cl)$—of all goods within R&D classes (exception made for the Low R&D products). However, other countries in South-East Asia feature for exporting high R&D goods, such as the Philippines in 2017 (see also Figure S4 of SM). East Europe and Central Asia (E. Europe & C. Asia), Latin America and the Caribbean (LAC), Middle East and North Africa (MENA), and Sub-Saharan Africa countries mainly export Medium-Low R&D goods, while importing products in the Medium-High and High R&D classes (see Fig. S3). Countries as Brazil, Egypt, Iran, Nigeria, and the Russian Federation are representative of this phenomenon (see Figs. 2b and 2c). Further information about the differences in the global export and import baskets can be found in Figure S4, which shows the composition of countries’ (and regions’) export and import baskets as a function of the R&D intensity of the traded products. In particular, we point out that countries in the OECD (e.g., Germany, Japan, and the USA) and South-East Asia (such as China and the Philippines) present relevant shares in their export basket in Medium-High and High R&D classes with reference to the year 2017.

Figures 2d and 2e show the traded dollars divided by the population (which is a proxy of the country size^22^) of the nation at hand. In these panels, we focus on the results regarding the products in the Medium-High class because this class includes the most traded volume of dollars in 2017 (Figs. S3, S5, and S6 in SM show the results for the other R&D classes and regions). Firstly, we notice that the dollars per capita are more homogeneously distributed for import than for export; this is in line with Fig. S5 of SM, especially for the products in the High R&D class. The OECD countries (e.g., Germany, Italy, Japan, and the USA) traded the highest amount of dollars per capita in 2017, especially in the Medium-High products. Meaningfully, considering its large population, China showed the highest per capita export-volume (in dollars) of Medium-High R&D goods among the BRIC countries (i.e., Brazil, the Russian Federation, India, and China) in 2017. Comparing Figs. 2d and 2e, China has larger per-capita export volume than in import. Conversely, Brazil, Colombia, the Philippines, and the Russian Federation present the opposite behaviour (and the per capita import is larger than the export one). Sub-Saharan countries (as Nigeria) exhibit the lowest per capita export and import volumes for the goods in the Medium-High R&D class. Figures S5 and S6 show the results for the other R&D classes for both countries (Figure S5) and regions (Fig. S6) in 2017. From the export point of view, reading the results from low to High R&D classes, countries in LAC (e.g., Brazil) decrease their ranking (in terms of dollars per capita) with respect to the others regions, while South-East Asia nations (e.g., China and India) increase it.

#### R&D embedded in countries export and import baskets

To study the R&D intensity embedded in countries’ export and import baskets, we define the country specific weighted share matrix **S**, whose elements are:

2 $$\begin{aligned} S(c,p)=\frac{D(c,p)}{\sum _p D(c,p)} \end{aligned}$$

where the matrix **S** is sub-indexed as ${{\textbf {S}}}_{{{\textbf {exp}}}}$ or ${{\textbf {S}}}_{{{\textbf {imp}}}}$ whether it refers to export and import baskets (correspondingly, the matrix **D** reads ${{\textbf {D}}}_{{{\textbf {exp}}}}$ and ${{\textbf {D}}}_{{{\textbf {imp}}}}$). The entry $S_{exp}(c,p)$ ($S_{imp}(c,p)$) reports the share of product *p* in the export (import) basket of country *c*. Recalling the vector **r**, containing the R&D intensity associated with each product, the R&D embedded in export (RDE) and import (RDI) of country *c* is defined as:

3 $$\begin{aligned} RDE(c)&=\sum _p S_{exp}(c,p)\cdot r(p), \end{aligned}$$

4 $$\begin{aligned} RDI(c)&=\sum _p S_{imp}(c,p)\cdot r(p) . \end{aligned}$$

Since the rows of the matrices ${{\textbf {S}}}_{{{\textbf {exp}}}}$ and ${{\textbf {S}}}_{{{\textbf {imp}}}}$ sum to 1, the RDE and RDI values range between the minimum and maximum of the vector **r** (see Table 1). The value of the RDE (RDI) of a country depends on the composition of its export (import) basket, encoded in the matrix ${{\textbf {S}}}_{{{\textbf {exp}}}}$ (${{\textbf {S}}}_{{{\textbf {imp}}}}$), and the R&D content of the traded items (in the vector **r**). If the RDE of a country is larger than 0.052 (i.e., the mean value of the vector **r**), then a notable share of its export basket is composed of High and Medium-High R &D products (the same reasoning holds for the RDI).

**Figure 3:**
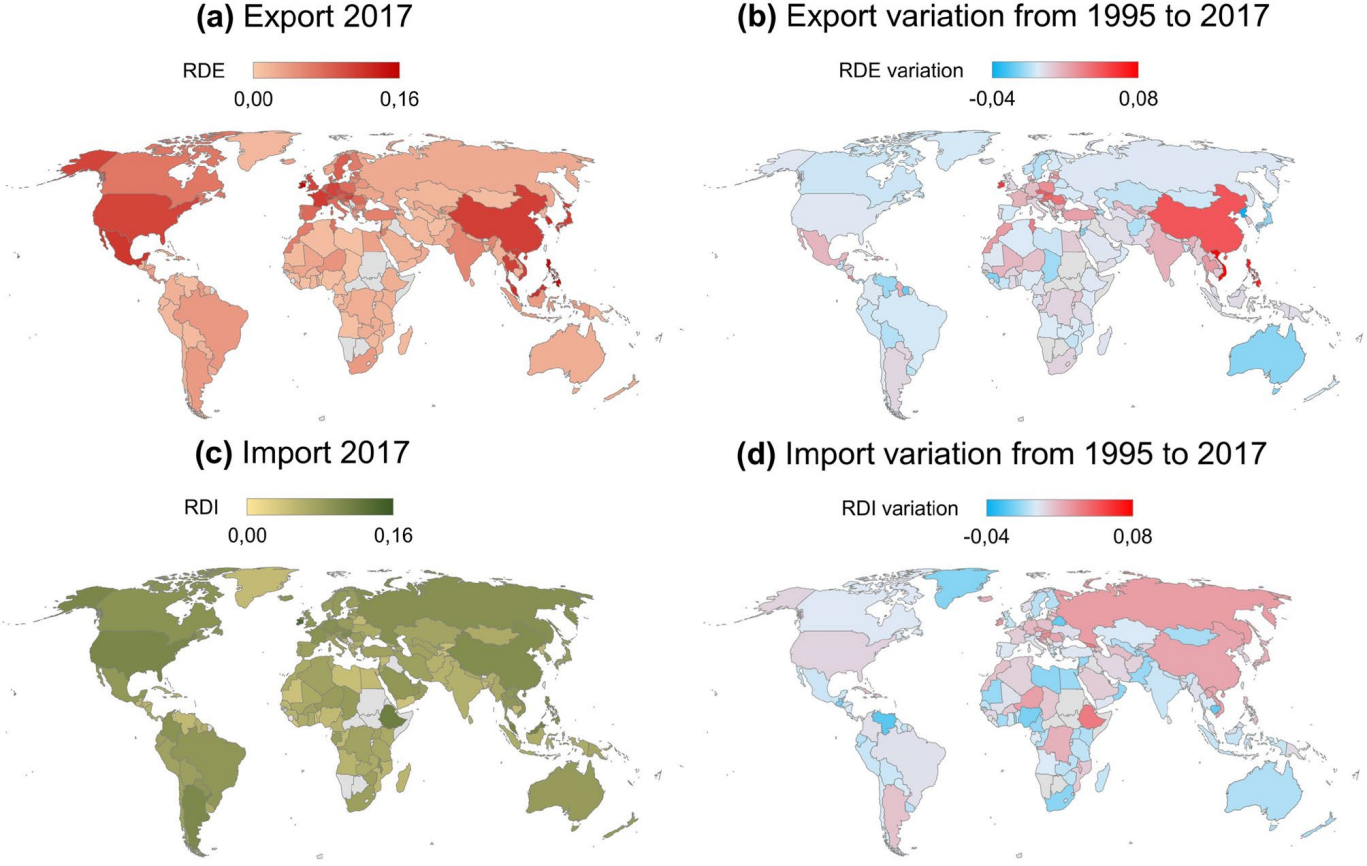
Research and Development embedded in the export (RDE, Eq. 3) and import (RDI, Eq. 4) baskets of countries worldwide. Panels (**a**) and (**c**) describe the geographical distribution of these indices for 2017 for the export and import baskets, respectively. Maps in panels (**b**) and (**d**) report the variation between 1995 and 2017 in RDE and RDI for each country, respectively. A positive variation means that the R&D embedded in the export or import basket of the country at hand has increased, while a negative value means that it has decreased. In all panels, countries where information is missing are in grey. The limits of the colour scales have been chosen to enhance the comparison between panels. The Figure is produced using Excel 2019.

Figure 3 maps the RDE and RDI values around the globe in the year 2017 (Figs. 3a and 3c), also highlighting their variation between 1995 and 2017, the first and last years of our time window (Figs. 3b and 3d).

Focusing on the export side (Fig. 3a), the largest RDE values are found in OECD countries in East Asia (Japan and South Korea), North America, and West Europe. Other noteworthy countries for their RDE are South-East Asian ones, such as China, the Philippines, and Vietnam. Countries in the remaining world regions exported products with lower R&D intensity than the previously cited countries, thus exhibiting a small RDE value (especially Sub-Saharan countries). Looking at the variations instead (Fig. 3b), South-East Asian countries notably enlarge the R&D intensity in their export basket during the period of analysis. This fact is particularly true for China, the Philippines and Vietnam, with the exception of North Korea which, conversely, shows a decrement in its RDE. Regarding the OECD countries, nations in Central and Western Europe (e.g., Czech Republic, Ireland, and Slovakia), Mexico, and Turkey present a rise in RDE; while Australia decreases its RDE of around 0.01.

On the import side (Fig. 3c), the highest values per region can be found in Ireland and the USA (OECD); Russian Federation (East Europe and Central Asia); China (South-East Asia); Eritrea (Sub-Saharan Africa); and Argentina (LAC). All the other countries in the world display an RDI of about 0.06. Regarding the changes in countries’ RDI (Fig. 3d), China and the Russian Federation show the highest RDI increase in East Europe and Asia. MENA and Sub-Saharan countries exhibit different variations; for example, Ethiopia and Nigeria increase their RDI, while Libya and South Africa decrease it. LAC countries show little variations, except for Argentina and Venezuela: while the former increases its RDI value, the latter significantly reduces it. OECD countries generally present a small-size rise in their RDI (e.g., Germany and the USA). The most sizeable increments are recorded in Japan and Eastern European countries (such as the Czech Republic and Slovakia). However, Australia, Finland, Mexico, and Sweden diminish their RDI between 1995 and 2017.

Figure S7 displays the trajectories of countries in the RDI-RDE plane from 1995 to 2017, showing that the majority of countries import products with higher R&D intensity than the exported goods (i.e., RDI>RDE). Among the highlighted countries, Germany, Japan, and the USA show RDE>RDI, while Brazil, Iran, and the Russian Federation present RDI>RDE for the whole time period. China and the Philippines exhibit a huge increment in their RDE.

**Figure 4:**
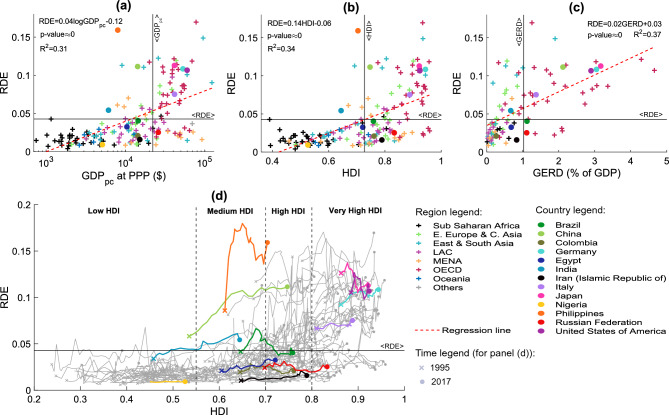
Comparison between countries’ RDE values and their per capita Gross Domestic Product at Purchasing Power Parity in constant 2017 dollars ($\hbox {GDP}_{\text {pc}}$ at PPP, panel (**a**)), Human Development Index (HDI, panel (**b**)), and Gross Expenditure in Research and Development as a percentage of GDP (GERD, panel (**c**)) for the year 2017. The colour of the plus signs refers to the organisation/geographical region the country belongs to according to Sachs et al.^49^. Filled dots highlight the 13 countries taken as examples throughout the text. Panels (**a**)–(**c**) are accompanied by the statistical description of the regression lines (dashed red lines). Here, the thick black lines mark the average values of the variables. Panel (**d**) shows the trajectories in the RDE-HDI plane for all countries in analysis (grey thin lines), highlighting those of Brazil, China, Colombia, Germany, Egypt, India, Iran, Italy, Japan, Nigeria, the Philippines, the Russian Federation, and the United States of America, as referred in the text (coloured lines). In this panel, the dashed black lines bound classes of HDI values as defined by the United Nations^50^. The Figure is produced with MATLAB R2020b.

The RDE and RDI report information regarding the R&D embedded in countries’ export and import baskets, which can be interpreted in light of the socio-economic conditions of countries. To better contextualize this information, we thus compare the proposed indices with the per capita Gross Domestic Product at Purchasing Power Parity ($\hbox {GDP}_{\text {pc}}$ at PPP), the Human Development Index^50^ (HDI), and the Gross Expenditure on Research and Development (GERD), expressed as a percentage of the Gross Domestic Product. While the GDP is purely an economic indicator, the choice of the HDI is reasoned by its mathematical formulation. In fact, the HDI combines life expectancy, years in school, and the Gross National Income of the citizens of a country and thus provides a definition of development that overcomes the economic well-being^50^. Using the GERD instead, allows us to frame the indicators with respect to the political economic choices of countries. Figure 4 shows the comparison between the RDE and the previously cited indicators (Figs. 4a–4c). In the plots, we define four quadrants as delimited by the average values of the quantities along the axes. In general, we observe that as the $\hbox {GDP}_{\text {pc}}$ and HDI increase (Figs. 4a and 4b), the RDE increases too. In fact, the majority of countries lie in the first and third quadrants. Therefore, in line with previous literature^13,30^, we speculate that in order to export high R&D products, countries must achieve high level of income and instruction (please see Figures S8 and S9 of SM showing the scatter plots of the RDE values with the number of researchers per million of inhabitants). Some countries featuring low $\hbox {GDP}_{\text {pc}}$ and HDI as Mali—MLI, Nigeria—NGA, Senegal—SEN, and Germany—DEU, Japan—JAP and the USA (for those with high $\hbox {GDP}_{\text {pc}}$ and HDI) exemplify this trend (see, Fig. S8). However, these quantities can explain at most 37% of the information contained in the RDE, and a deeper reflection is needed to contextualize the outliers. On the one hand, in the second quadrant, we find countries such as the Philippines and Vietnam—VNM, which export high R&D goods, but have low $\hbox {GDP}_{\text {pc}}$ and HDI values (also see Fig. S8). This might possibly be related to the specialization in their economic activity^51,52^. On the other hand, in the fourth quadrant, we find countries exporting low R&D products despite their above-average $\hbox {GDP}_{\text {pc}}$ and HDI. This behaviour appears to characterize economies relying on natural resources, such as fossil fuels, that have low R&D intensity values (e.g., the Russian Federation, Qatar—QAT, and the Union of Arab Emirates—ARE ^53^), but can be very economically productive (see Figure S8).

Figure 4c compares the RDE with the GERD, providing a match between the R&D efforts of countries (proxied by the GERD) and what they trade (the RDE). The positive slope of the regression line supports the idea that as the countries’ efforts in R&D increase, the R&D embedded in its exports also grows. However, notable differences occur. Specifically, Mexico—MEX, Vietnam—VNM, and Thailand—THA export high R&D products while presenting low GERD (please, see Figure S8). The comparison between the RDI values and the herein-used socio-economic indicators shows a weaker relationship than what stated for the RDE ones (please refer to Figs. S10 and S11). It follows that the RDE and RDI (combining countries’ trade basket composition with the R&D intensity of the exchanged products) provide complementary information with respect to the $\hbox {GDP}_{\text {pc}}$, HDI, and GERD, which can contribute to unveiling new and unexpected aspects of the country-specific propensity to economic well-being, development, and innovation.

It is interesting to investigate the evolution in time of countries’ development (approximated with the HDI) and the R&D content embedded in the nations’ trade baskets^3,5–15^. Figure 4d shows the paths of countries in the RDE-HDI plane between 1995 and 2017 (the trajectories in the RDI-HDI plane are in panel (d) of Fig. S10). Generally, the countries’ HDIs increase in time, and so do their RDE values, thus confirming the outcome of the scatter plots in Figs. 4a–4c. However, within this general trend, different behaviours emerge. Countries with low HDI (e.g., Nigeria in the plot) have a small and steady RDE because more than 90% of its export basket is composed by Medium-Low R&D products all along the period of analysis (please see Fig. S12), and their RDI is larger than RDE (one can compare Fig. 4 with Fig. S10). As the HDI increases, the behaviour of countries diversifies. The majority of medium-to-high HDI countries have below-average RDE values, such as Egypt and the Russian Federation, confirming the role of natural resources in their development path (we recall that the HDI is a composite indicator where the Gross National Income is included). In fact, although having recorded an increment in their HDI, the Russian Federation presents a quasi-constant RDE in the period of analysis and an increasing RDI (please see Fig. S10). We realize this behaviour is typical of fuel-exporting economies, such as Colombia and the Russian Federation^53^, whose import baskets mainly comprise high R&D products (please see Fig. S13 of SM). Egypt stands as an exception to this pattern since the country improved the composition of its export basket in time, as the Fig. S12 of SM shows in terms of R&D classes. Egypt’s trend is even more evident for some others medium-to-high HDI countries, such as China and the Philippines. Among these countries, the clearest path is the one of China, which has passed from a low HDI to a high HDI value between 1995 and 2017 while changing its export baskets from Medium-Low to Medium-High and High R&D products. This transition of the export basket is accompanied by an increasing GERD and researchers numbers (please, see Fig. S14). Developed economies (e.g., Germany, Japan, and the USA) are in the top right region of the figure. These economies present high RDE, with little variations in time. Japan shows an increment in terms of HDI while reducing (increasing) its RDE (RDI, see Fig. S9): the shares of exports in High R&D products reduces in favor of their imports (see Figs. S12 and S13), despite growing investment in R&D (Fig. S14). Among developed economies, Germany presents the highest growth in terms of RDE: during the period of analysis, High R&D products gained shares within its export basket while reduced were those in Low and Medium-Low R&D products (Fig. S12).

### From R&D intensity: gaining insights on environmental performances

In the following, we present two possible applications of the newly introduced indices to investigate on countries’ environmental performances. Our example grounds on recent literature investigating the innovation-sustainable development nexus^34–42^, and the capability of the RDE in providing new information in terms of countries’ development and R&D activities. Our analysis is two-fold: the first part focuses on the products with environmental benefits (which Mealy et al.^54^ linked to a reduction of the $\hbox {CO}_2$ per capita emissions), and the second part refers to the $\hbox {CO}_2$ emissions embedded in the traded goods.

#### Green products

Considering that R&D and innovation are crucial to address the green transition^37,39^ and to advance economic performance^6,10,15^, we analyse the market of the products with environmental benefits (so defined *green products*^54,55^) from an R&D viewpoint. A possible definition of green products includes those goods measuring, preventing, limiting, minimizing, and correcting the environmental damages to water, soil, and air, also dealing with waste-, noise-, and ecosystems-related issues^56^. However, the identification of green products is a challenging task. Firstly, many products can be used for several purposes, going beyond those uses especially targeting environmental issues^54,57^. Secondly, the products’ environmental performance can change with technological progress; thus, any green product list has to be updated periodically^54,58^. Since an internationally accepted list of green products does not exist^55,59^, we consider the green products inventory proposed by Mealy et al.^54^. To the best of our knowledge, this list is the most detailed and recent catalogue of green products obtained by combining data from three international institutions (i.e., OECD^58,60^, World Trade Organisation^61^, and Asia-Pacific Economic Cooperation^62^).

**Figure 5:**
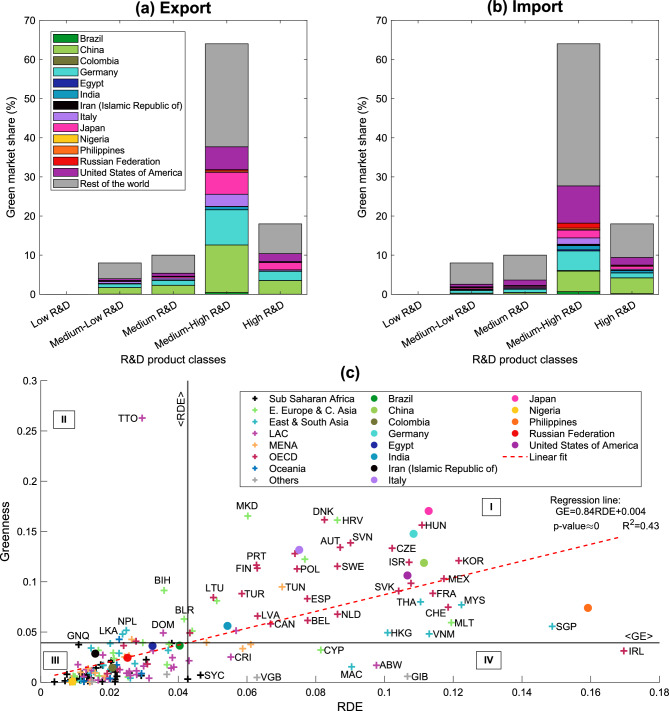
R&D description of the green products market in 2017. Panels (**a**) and (**b**) describe the green market in terms of R&D classes (see Eq. (6) in the Methods section). Panel (**c**) shows the scatter plot between the greenness (GE) and the R&D embedded in countries’ export basket (RDE, Eq. (3)). In panel (**c**), the vertical and horizontal black solid lines represent the mean values of the quantities along the axes. The colour of the plus signs refers to the organisation/geographical region the country at hand is included in according to Sachs et al.^49^. Filled dots highlight the 13 countries taken as examples throughout the text. The ISO 3-alpha code is used to tag countries, and correspondence is given in Tables S1 and S2 of the SM. The Figure is produced with MATLAB R2020b.

In this work, we study the monetary fluxes associated with green products (called *green market*) in terms of R&D intensity. We define the *greenness* (GE) of country *c* as the ratio between the dollars country *c* exports in green products and the total volume of dollars exported by country *c*. In mathematical terms: $GE(c)=\sum _{p\in G} D_{exp}(c,p) / \sum _p D_{exp}(c,p)$, where *G* is the set of green products defined in Mealy et al.^54^. Figure 5 summarizes our main results. Figures 5a and 5b show that the largest shares of dollars in the green market are traded through the Medium-High and High R&D products (these classes contain the largest number of green products, see Figure S15 of SM). Moreover, Figs. 5a and 5b display the green market share owned by each country in 2017 for each R&D class, i.e., applying Eq. (1) only to the green products (see “Data and methods”). In general, OECD and South-East Asian countries are the protagonists in the green market landscape, on both the export and import sides, while countries in LAC, MENA and Eastern Europe, and Central Asia are net-importers of green products (please, see Fig. S16 of SM). Specifically, China, Germany, Italy, and Japan own significant shares in the green export market, confirming previous studies^54,55^. Conversely, Iran, the Russian Federation, and USA are net-importers of green products.

Figure 5c places the countries in the plane identified by the greenness and RDE of their export baskets (see Eq. 3). Countries can be characterized by considering their position in the plot in the four quadrants, as identified through the average values of both greenness and RDE. The majority of countries are in sectors I and III. In general, both developed countries in the OECD (such as Germany, Italy, Japan, and the USA) and developing countries in South-East Asia (such as China and India) appear in sector I. Developing countries in LAC (e.g., Brazil) and Sub-Saharan Africa (e.g., Nigeria), and economies in transition (e.g., the Russian Federation) figure in region III. Through a linear regression (red dashed line), we observe that the greenness is significantly related to the research and development embedded in the countries’ export baskets (the $\hbox {R}^2$ is 0.43). The regression results are similar for the other years in our time window (please, see Fig. S17 of SM).

#### $\hbox {CO}_2$ export intensity and R&D

**Figure 6:**
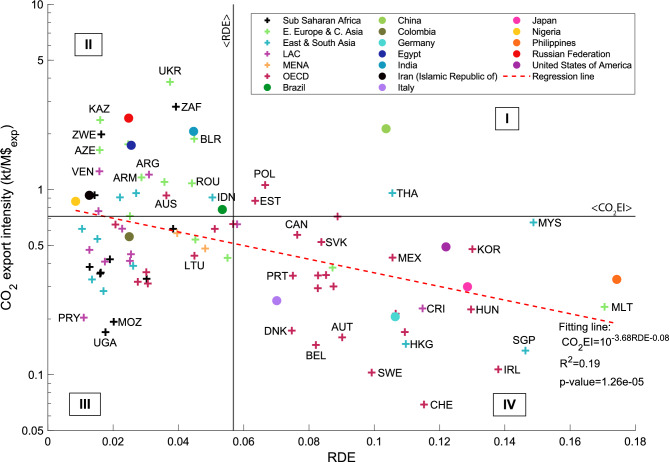
Position of countries in the plane $$\hbox {CO}_2$$ export intensity (measured in kilotons, kt, of $$\hbox {CO}_2$$ over millions of dollars exported, M$$$_{exp}$$, in brief, $$\hbox {CO}_2$$EI) versus RDE (see Eq. (3)). This plot refers to 2004 because $$\hbox {CO}_2$$ data are from Davis et al.^46^ that published data that year. Moreover, only single countries, and not agglomerates of countries, were considered in this analysis. The fitting line is drawn considering a function $$CO_2EI=10^{aRDE+b}$$ (notice that the y-axis is in logarithmic scale). The colour of the plus signs refers to the organisation/geographical region the country at hand is included in according to Sachs et al.^49^. Filled dots highlight the 13 countries taken as examples throughout the text. The ISO 3-alpha code is used to tag countries, and correspondence is given in Tables S1 and S2 of the SM. The Figure is produced with MATLAB R2020b.

We use the R&D intensity framework to describe the $\hbox {CO}_2$ content in countries’ exports, trying to gain insightful information about the nexus between trade and environmental outcomes. $\hbox {CO}_2$ emissions are a good proxy of the environmental impact of countries considering its Global Warming Potential and its anthropogenic emissions from the industrial sectors^46,47,63^. To perform such analysis, we consider the $\hbox {CO}_2$ emissions embedded in a country export basket divided by the dollars the country exports, thus defining a country-specific $\hbox {CO}_2$ export intensity ($\hbox {CO}_2$EI). By introducing this quantity, we can compare—in terms of $\hbox {CO}_2$ emissions due to the production of the exported goods and dollars in export—economies of different size (e.g., China and Colombia). Then, we relate the RDE (Eq. (3)) with the $\hbox {CO}_2$ export intensity. Our analysis is shaped by the availability of $\hbox {CO}_2$ emissions data. Firstly, the analysis is conducted by taking the data referring to 2004 as published by Davis et al.^46^. Figure 6 reports the position of the countries in this plane using the 2004 data. Generally, as the countries’ RDE increases, the associated $\hbox {CO}_2$ export intensity decreases. The exponential interpolation with the function $CO_2EI=10^{aRDE+b}$ supports the previous observation; in fact, the parameter *a* is negative and statistically significant (*p*-value < 0.01). Considering the average values of the quantities along the axes, as done in panel (c) of Figure 5, we define four sectors of the graph. Countries showing large RDE while emitting a notable amount of $\hbox {CO}_2$ per exported dollar are in sector I. China, Estonia, Poland, and Thailand featured in this sector in 2004. Sector II contains those countries exporting products with small R&D and presenting high $\hbox {CO}_2$ export intensity. In this sector, in 2004, we mainly find countries in East Europe and Central Asia, LAC, and MENA (e.g., Egypt and Iran) region, most of them relying on the export of fossil fuels, such as Azerbaijan, the Russian Federation, and Venezuela^53^. In sector III, we see countries exporting simple products and having a low $\hbox {CO}_2$ export intensity. Here, LAC countries (e.g., Paraguay, PRY) and the majority of African countries (e.g., Mozambique—MOZ and Uganda—UGA) appeared in 2004. Sector IV includes countries presenting large RDE and with low $\hbox {CO}_2$ export intensity. Here, developed OECD (such as Germany, Italy, Japan, and the USA) and developing South-East Asia (i.e., Hong Kong—HKG, the Philippines, and Singapore—SGP) economies^53^ figured out in 2004.

Secondly, we extend the analysis including all the years in our time window by inferring the $\hbox {CO}_2$ emissions embedded in the export of countries starting from the data from the Global Carbon Budget^64^ and the World Development Indicators^65^, also enlarging the geographical availability of the data in Davis et al.^46^ (see Data and methods). Figure S18 in SM reports the value (and *p*-value ranges) of the parameter *a* of the exponential fit. One can see that: (i) the parameter *a* is always negative, meaning that the $\hbox {CO}_2$ export intensity decreases as the RDE of countries increases; and (ii) this parameter is almost always significant, i.e., the associated *p*-value is less than 10%.

Figure S19 of SM shows the same analyses reported above, considering the greenness instead of the RDE. The RDE is a better parameter to describe the $\hbox {CO}_2$ export intensity than the greenness, confirming the R&D centrality in the green transition and decarbonization process.

## Discussion and conclusions

In this work, we develop a matching algorithm that assigns to each product a country trades (as expressed through the Harmonized System categorisation) the R&D intensity of the industrial sector it is included in. Using this information, we are able to study international trade and countries’ trade baskets from an R&D perspective. We summarize the R&D content embedded in countries’ export and import baskets in two indices: the RDE and RDI, respectively. Through these indices, we show that countries differentiate—in terms of R&D intensity embedded in the exchanged goods—from each other mainly in the export basket than in the import basket. Countries with the highest R&D embedded in their export basket are in the Organisation for Economic Co-operation and Development (e.g., Germany, Japan, and the USA) and South-East Asia (i.e., China, the Philippines, and Vietnam). The latter region presents notable growth in the export of Medium-High and High R&D products. The proposed indicators combine industry-related R&D data with the composition of countries’ trade baskets, providing another benchmark of comparison for economics and development. Comparing the RDE and RDI with the per-capita GDP, HDI, and GERD, we show that our indices provide complementary information to standard measures of development and R&D performances. Relying on our approach, we described the development trajectories of countries in the RDE-HDI plane, highlighting that medium-to-high HDI countries may exhibit diverse behaviours, as exemplified by the trajectories of China and the Russian Federation, with reasons to be found in their reliance on natural resources and key-economic sectors. Conversely, nations with small and very high HDI present almost-stable export basket composition (in terms of R&D classes).

With the aim of providing examples of applications of such indicators to unveil further countries’ performative characteristics, also considering the role of R&D in achieving sustainable development^34–42^, we apply the proposed indices (especially the RDE) to describe countries’ environmental performances. In particular, we focus on the trade of green products, showing that these products can be generally flagged as being part of the Medium-High and High R&D classes. Furthermore, we show that the share of green products in countries’ export baskets (i.e., what we defined as *greenness*) correlates—in a statistically significant way—with the corresponding RDE values. Lastly, we find out that at parity of monetary volume exported, smaller $\hbox {CO}_2$ emissions can be associated with countries with larger shares of high-R&D products.

Our novel perspective presents some drawbacks that arise from the input data, which we attempt to tackle and hereby further discuss. The R&D classification of industrial sectors proposed by Galindo-Rueda et al.^32^ is calculated on a small subset of countries. As stated by the authors^32^, an ideal R&D taxonomy should comprehend all economies in the world, and data availability is the bottleneck of this methodological approach to categorisation. On the back of the sensitivity studies performed by the authors on the R&D taxonomy, in the Supplemental Material, we show that to include other countries’ data, stable RDE and RDI are found (please refer to the SM and Galindo-Rueda et al.^32^ for further details). Another limitation that comes from data availability relates to the aggregation of products into industrial activities. Thus, all the products in the same industry are associated with the R&D intensity value (see Figure 1). This fact leads to a loss of information, with the RDE and RDI unable to distinguish which product is responsible for the economic output in that given industry (please refer to Data and methods section and SM). To minimize this issue, this work uses the most detailed industrial sector R&D taxonomy, which is the one proposed by Galindo-Rueda et al.^32^, and future industrial categorisation can help improve the resolution of the algorithm at the product level.

This research is a first step toward combining countries’ trade performances, innovation, investment choices and, no less, environmental performances. Since framed within the cross-section of disciplines, we address future research towards relating products’ R&D intensities with their sophistication as defined within the well-known economic complexity theory^26–29^. Moreover, our work paves the way to investigate the R&D-Environment-Development nexus considering the composition of countries’ trade baskets as the starting point. While this work grounds on regression analyses to analyse this link, future work can address the causal relationships within this nexus, trying to understand whether the R&D embedded in the exchanged goods is one of the leading factors affecting sustainable development. In this direction, we will consider the comparison of the RDE with more sustainability-related indices (e.g., the Environmental Performance Index^66^ and the Sustainable Development Goals Index^67,68^). Eventually, one can investigate the relationship between the trade of green products, the added cost of low-carbon transition (i.e., the green premium^69,70^, which can also be considered to improve categorization of the green products), and RDE-RDI values of countries.

## Data and methods

### Trade data

The composition (in terms of products, economic values and quantities) of the countries’ export and import baskets in the period 1995–2017 is obtained from the BACI-CEPII dataset^33^. This information is encoded in two matrices, named ${{\textbf {D}}}_{{{\textbf {exp}}}}$ and ${{\textbf {D}}}_{{{\textbf {imp}}}}$ for the export and import data, respectively. The element $D_{exp}(c,p)$ ($D_{imp}(c,p)$) details the dollars country *c* traded through the export (equivalently, import) of product *p* in a given year. The period of analysis moves from 1995 to 2017. Products are classified according to the Harmonized System Codes 1992 (HS-1992) at the 6-digit level. To filter away the noise due to small fluxes, we only include countries presenting a total export flux ($\sum _p D_{exp}(c,p)$) larger than 0.001% of the monetary value of the whole international trade (i.e., $\sum _{c,p} D_{exp}(c,p)$). Time series are defined considering countries with full records of data within the time frame of analysis (i.e., 1995-2017).

### R&D intensity data

The Research and Development data (R&D data) are retrieved from the Organisation for Economic Co-operation and Development (OECD), as described by Galindo-Rueda et al.^32^. In their work, the authors computed an R&D intensity value for each industry sector identified by the International Standard Industrial Classification revision 4 (ISIC rev 4). The authors calculated the R&D intensity of an industrial sector (*I*(*i*)) considering aggregated data from 29 countries (Australia, Austria, Belgium, Canada, Czech Republic, Denmark, Estonia, Finland, France, Germany, Greece, Hungary, Ireland, Italy, Japan, Mexico, Netherlands, Norway, Poland, Portugal, Republic of Korea, Singapore, Slovakia, Slovenia, Spain, Sweden, Taiwan, the United Kingdom, and USA) in 2011 as^32^:

5 $$\begin{aligned} I(i)=\frac{\sum _c E_{R \& D}(c,i)}{\sum _c GVA(c,i)}, \end{aligned}$$

where $E_{R \& D}(c,i)$ (*GVA*(*c*, *i*)) is the expenditure in R&D (Gross Value Added) of country *c* in the industry *i* in dollars at Purchasing Power Parity^32^. Considering the conversion key from products to industry, i.e., from HS-1992 to the ISIC rev. 4 available at https://www.oecd.org/sti/ind/ConversionKeyBTDIxE4PUB.xlsx, we are able to associate an R&D level to any product (expressed by 6-digit codes in the Harmonized System) a country exports or imports. Table 2 reports the R&D classes, with associated ranges of R&D intensity, number of products, and product industries contained in the class at hand.

**Table 2:** R&D classes description.

R&D class	R&D intensity range^32^	Number of products in 6-digit code	Industries
High	>0.20	361	Air and spacecraft machinery; pharmaceutical goods; scientific research and development instruments; computer, electronic and optical products
Medium-High	[0.05, 0.20]	1591	Weapons and ammunition; motor vehicles, trailers and semi-trailers; medical and dental instruments; chemicals and chemical products; electrical equipment; railroad, military vehicles and transport
Medium	[0.018, 0.05]	869	Rubber and plastic products; building of ships and boats; basic metals; repair and installation of machinery and equipment
Medium-Low	[0.005, 0.018]	1881	Textiles; leather and related products; paper and paper products; food products, beverages and tobacco; wearing apparel; coke and refined petroleum products; mining and quarrying; wood and products of wood and cork
Low	$\le$0.005	300	Agriculture, forestry and fishing

Notice that, Galindo-Rueda et al.^32^ provided an industry-specific R&D intensity value that does not depend on the single country characteristics. As introduced in the Discussion and Conclusions section, the subset might still be considered not large enough to represent the diversity of countries in the world. Yet, to the best of our knowledge, the R&D taxonomy of industrial sectors proposed by Galindo-Rueda et al.^32^ is the most recent and the only existing one that is centered on R&D, also being in line with previous technology-based classification (the Eurostat technology classification in NACE rev. 2 and the OECD technology classification in ISIC rev. 3). This R&D classification proved to be robust to several sensitivity tests. In particular, the classification has been found stable with the inclusion of China’s data—even if provided at a lower resolution (i.e., with larger industry aggregation); and also considering other possible definitions of the intensity value (see Eq. (5)). Against this background, sensitivity tests on our newly introduced categorisation have been performed, with stable results found and here shown in the SM. We refer the interested reader to Galindo-Rueda et al.^32^ for further details.

### Green market share

Recalling Eq. (1), the green market share of country *c* for the green products in the R&D class *cl* (*GM*(*c*, *cl*)) is computed as:

6 $$\begin{aligned} GM(c,cl)=\frac{\sum _{p\in G, p\in cl} D(c,p)}{\sum _{c, p\in G} D(c,p)} \cdot 100, \end{aligned}$$

where *G* is the set of green products defined by Mealy et al.^54^. As for the matrix **M**, **GM** is sub-indexed as ${{\textbf {GM}}}_{{{\textbf {exp}}}}$ or ${{\textbf {GM}}}_{{{\textbf {imp}}}}$ depending on **D** reads ${{\textbf {D}}}_{{{\textbf {exp}}}}$ or ${{\textbf {D}}}_{{{\textbf {imp}}}}$.

### $\hbox {CO}_2$ data

$\hbox {CO}_2$ emissions embedded in countries’ export baskets for the year 2004 come from the work of Davis et al.^46^. To extend the analysis to all the years between 1995 and 2017, the $\hbox {CO}_2$ emissions embedded in the export ($\hbox {CO}_2^{exp}$) are estimated starting from the total amount of $\hbox {CO}_2$ a country emits (i.e., territorial $\hbox {CO}_2$ emissions). Data of territorial $\hbox {CO}_2$ emissions are from the Global Carbon Budget^64^ for the period 1995-2017. To estimate the $\hbox {CO}_2$ embedded in the export of countries, the Merchandise Export (MerchExp, i.e., the export of goods, not services) and Gross Domestic Product (GDP), both in dollars, are considered for all countries in the world and the years between 1995 and 2017 from the World Development Indicators^65^. The $\hbox {CO}_2$ emissions in the export of country *c* in year *t* ($CO_{2}^{exp}(c,t)$) are estimated as:

7 $$\begin{aligned} CO_{2}^{exp}(c,t)=CO_{2}^{terr}(c,t) \frac{MerchExp(c,t)}{GDP(c,t)}, \end{aligned}$$

where $CO_{2}^{terr}(c,t)$ is the territorial $\hbox {CO}_{{2}}$ of country *c* in the year *t*. The ratio *MerchExp*(*c*, *t*)/*GDP*(*c*, *t*) represents the fraction of the total GDP of country *c* due to the export of goods. Applying this ratio to the territorial $\hbox {CO}_2$ of the country at hand, we estimate the $\hbox {CO}_2$ embedded in the export of countries. Figure S20 of SM displays the comparison between the $\hbox {CO}_2$ in export estimated with Eq. (7) for 2004 with the $\hbox {CO}_2$ embedded in the export of countries from the paper of Davis et al.^46^. Figure S20 shows that the two datasets match well; thus, we use Eq. (7) to estimate the $\hbox {CO}_2$ emissions embedded in export between 1995 and 2017. Since the number of countries in the Global Carbon Budget^64^ and World Development Indicators^65^ datasets is larger than that in Davis et al.^46^, we can extend the analysis to a wider set of countries.

## Supplementary Information

Supplementary Information.

## Data Availability

The R&D intensity values of each industry are from Galindo-Rueda et al.^32^. The trade data supporting this analysis come from the BACI-CEPII dataset^33^. The key to associate a product codified in the Harmonized System to the industry it is included in (using the ISIC nomenclature) is developed by the OECD and freely available at https://www.oecd.org/sti/ind/ConversionKeyBTDIxE4PUB.xlsx. The GDP, GDP per-capita, GERD, merchandise export, number of researchers, and population data are from the World Development Indicators^65^, HDI data are freely available at https://hdr.undp.org/data-center/human-development-index#/indicies/HDI, and territorial $\hbox {CO}_2$ data come from the Global Carbon Budget^64^. $\hbox {CO}_2$ emissions embedded in countries’ export baskets for the year 2004 are from the work of Davis et al.^46^.
