# Supplementary material for: An R&D perspective on international trade and sustainable development
Source: Sci Rep. 2023 May 17;13:8038. doi: 10.1038/s41598-023-34982-3 (PMC10192450; doi:10.1038/s41598-023-34982-3)
Supplement: Supplementary file 1 — Supplementary Information. [file 41598_2023_34982_MOESM1_ESM.pdf]

An R&D perspective on international trade and sustainable development. Supplemental Material

Lorenzo Costantini[1,*], Francesco Laio[1,+], Luca Ridolfi[1,+], and Carla Sciarra[1,+]

[1]Politecnico di Torino, DIATI, Turin, 10129, Italy
[*]lorenzo.costantini@polito.it
[+]these authors contributed equally to this work

ABSTRACT

This Supplemental Material (SM) details the R&D analyses on the international trade grouping the countries according to the seven world regions considered in Sachs et al.[1]: East Europe and Central Asia (E. Europe & C. Asia), East and South Asia, Latin America and the Caribbean (LAC), Middle East and North Africa (MENA), Oceania, countries in the Organisation for Economic Co-operation and Development(OECD), Others, and Sub Saharan Africa (note that the region "Others" groups all countries not included in any regions). Moreover, this document reports further details on the analyses presented in the main text, the linkages between the RDE and greenness (GE), countries' CO_2 emissions, and development trajectories (please, see the main text).

Supplemental figures

- Figure S1 displays the number of products as function of the R&D intensity.
- Figure S2 shows the time series of the five R&D classes in terms of traded dollars and market shares.
- Figure S3 presents the same results of Figure 2 in the main text with countries aggregated in regions according to the clustering in Sachs et al.[1] in 2017. Notice that to compute the values in the plot entails defining the matrix **MR**, whose element $MR(r,cl)$ represent the market share of the region r in the R&D class cl. The value is built by accounting for all of the countries falling in that region and the products in the given R&D class. In mathematical terms:

$$MR(r,cl) = \frac{\sum_{c\in r, p\in cl} D(c,p)}{\sum_{c,p} D(c,p)} \cdot 100 \tag{S1}$$

  where **D** is the matrix reporting in each cell the dollars country c exports (imports) in product p, named $\mathbf{D_{exp}}$ ($\mathbf{D_{imp}}$, please see the main text). Thus, the matrix **MR** is sub-indexed as $\mathbf{MR_{exp}}$ or $\mathbf{MR_{imp}}$ whether it refers to the export or import basket. This figure also reports, for the products in the Medium-High R&D class, the per-capita dollars exported and imported in these regions as a function of the population.
- Figure S4 reports the shares in countries' trade baskets as a function of the R&D intensity of the exchanged goods. The top panels refer to the countries, bottom panels to the regions in 2017.
- Figures S5 and S6 show the per-capita dollars exported and imported in each R&D class. Thus, Figure S5 complements Figure 2 of the main text and Figure S6 complements Figure S2 of this SM.
- Figure S7 displays the trajectories of countries in the RDI-RDE plane from 1995 to 2017 (see the main text for the definition of RDE and RDI).
- Figure S8 compares the RDE with the per capita Gross Domestic Product at Purchasing Power Parity (GDP_{pc} PPP), Human Development Index[2] (HDI), Gross Expenditure on Research and Development (GERD) expressed as a percentage of the GDP, and number per researchers per million of inhabitants (N_r). Countries are identified by their ISO 3-alpha code.
- Figure S9 shows the comparison between the RDE and RDI values vs the number of researchers per million of inhabitants (N_r).

- Figure S10 compares RDI with the Gross Domestic Product per capita at Purchasing Power Parity (GDP_{pc} PPP), Human Development Index[2] (HDI), and Gross Expenditure on Research and Development (GERD) expressed as a percentage of the GDP.

- Figure S11 reports the parameters of the fits between the RDE and RDI values versus the GDP_{pc}, HDI, and GERD ones.

- Figures S12 and S13 describe the export and import baskets, respectively, of the 13 countries used as examples throughout this work for the years 1995 and 2017.

- Figure S14 reports the time series of GERD and the number of researchers from 1995 to 2017. Please note that only countries with complete records are shown.

- Figure S15 shows the number of green products in each R&D class.

- Figure S16 shows the shares held by regions in the market of green products (called *green market*) for each R&D class. In mathematical terms, the green market share of region r in the class cl constructed only considering the green products ($GMR(r,cl)$) is:

$$GMR(r,cl) = \frac{\sum_{c\in r, p\in G, p\in cl} D(c,p)}{\sum_{c,p\in G} D(c,p)} \cdot 100, \quad \text{(S2)}$$

  where G is the set of green products identified by Mealy et al.[3]. As for the matrix **MR**, **GMR** can be sub-indexed as $\mathbf{GMR_{exp}}$ ($\mathbf{GMR_{imp}}$) whether **D** is $\mathbf{D_{exp}}$ ($\mathbf{D_{imp}}$).

- Figure S17 reports the slope, p-value, and R^2 of the fit between the RDE and greenness (GE, see the main text) in the period 1995-2017.

- Figure S18 shows the parameter a (and associated p-value ranges) between 1995 and 2017, considering the CO_2 export intensity (CO_2EI, please see main text) estimated with equation (7) of the main text, i.e., using data from the Global Carbon Budget[4] and the World Bank[5]. We here recall that the fitting function is $CO_2EI = 10^{aRDE+b}$.

- Figure S19 shows the comparison between the CO_2 export intensity and the greenness.

- Figure S20 provides information regarding the goodness of the estimation procedure of the CO_2 emissions embedded in countries' exports. The plot scatters the values of CO_2 embedded in countries' export baskets as given by Davis et al.[6] with those computed through the estimation procedure in Equation (7), thus using the data from the Global Carbon Budget[4] and the World Bank[5]. Values refer to the year 2004.

Tables S1 and S2 report the description of the ISO 3-alpha code used throughout the work to describe the countries in the figures.

Sensitivity analyses on the R&D data

As discussed in the main text, the industrial R&D taxonomy proposed by Galindo-Rueda et al.[7] is computed on a relatively small subset of countries, with the majority being OECD member states (27 countries out of 29, the remaining two countries are Singapore and Taiwan). In fact, Galindo-Rueda et al.[7] observed poor data availability to compute the R&D intensity for non-OECD countries. To study possible fluctuations of their proposed taxonomy, the Authors in[7] computed the R&D intensity values also considering China's data, despite their lower resolution (i.e., a larger product-industry aggregation) compared to that of the selected 29 countries. In doing so, the Authors noted that to consider such data in the definition of the taxonomy, the R&D intensity values decreased in two-thirds of industries, being "computer, electronics and optical products"; "transport equipment" and "chemicals and pharmaceuticals" the most affected from this decrement, because China has relevant Gross Value Added shares in these industries despite lower R&D efforts than the other considered countries. However, the inclusion of China only slightly changes the R&D classification of three industries: "textiles", which upgrades to Medium R&D industries from Medium-Low R&D; "computer, electronic and optical products", downgrading from High to Medium-High R&D; "other non-metallic mineral products" from Medium to Medium-Low R&D industries. Therefore, the class definition and the industry rankings remain stable in spite of China's data inclusion. As a result, it seemed reasonable to assume that the Galindo-Rueda et al.'s[7] taxonomy could be used at the world level, lacking of other available datasets.

However, following the aforementioned robustness checks, we also analysed possible fluctuations of the proposed indicators (i.e., the RDE and RDI) considering as input the R&D taxonomy including China's data. These values are hereby identified as

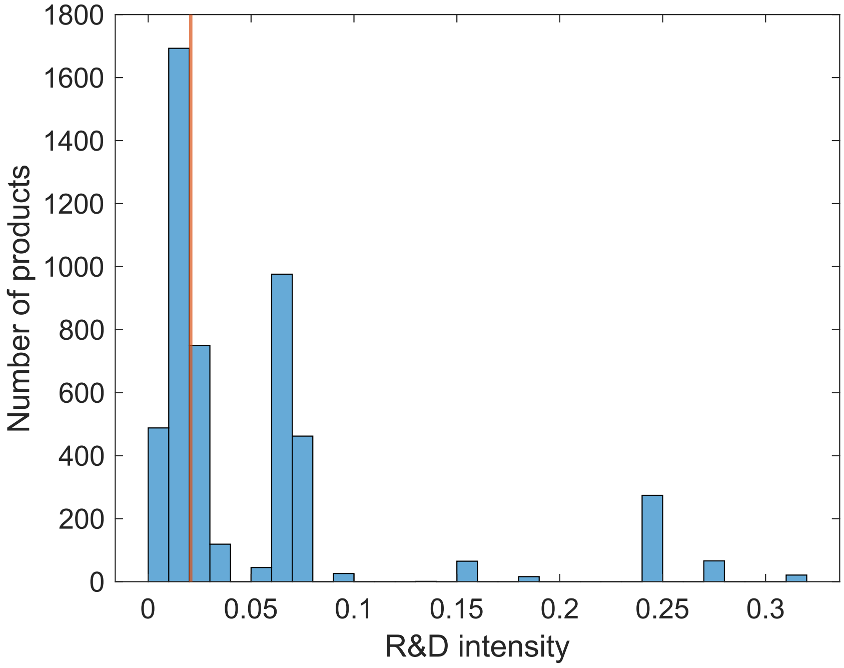

Figure S1. Number of products as a function of the R&D intensity. The vertical red line indicates the median value of the R&D intensity values embedded in the exchanged goods (please, see the main text). The Figure is produced with MATLAB R2020b.

RDE* and RDI*. Figure S21 shows (in the left panels) the RDE* and RDI* values for the countries considered in our work in the year 2017 (for comparison, we refer to Figure 3 of the main text), and the RDE-RDE* and RDI-RDI* correlation values in time (right panel). As we expected, the RDE* and RDI* values are lower than the RDE and RDI, respectively, but the results in terms of ranking are stable. In fact, the RDE* and RDI* present high Spearman's correlation values (i.e., rank-based correlation) with the RDE and RDI.

References

1. Sachs, J. *et al.* The sustainable development goals and covid-19. *Sustain. development report* **2020** (2020).
2. UNDP. Human development report 1990 (1990).
3. Mealy, P. & Teytelboym, A. Economic complexity and the green economy. *Res. Policy* 103948 (2020).
4. Friedlingstein, P. *et al.* Global carbon budget 2021. *Earth Syst. Sci. Data* **14**, 1917–2005 (2022).
5. WorldBank. World development indicator data sets. *Dataset* https://databank.worldbank.org/source/world-development-indicators.
6. Davis, S. J. & Caldeira, K. Consumption-based accounting of co2 emissions. *Proc. national academy sciences* **107**, 5687–5692 (2010).
7. Galindo-Rueda, F. & Verger, F. Oecd taxonomy of economic activities based on r&d intensity. *available at SSRN: https://www.oecd-ilibrary.org/content/paper/5jlv73sqqp8r-en* DOI: https://doi.org/https://doi.org/10.1787/5jlv73sqqp8r-en (2016).

Acknowledgements (not compulsory)

We thank Penny Mealy and Alexander Teytelboym for sharing with us the green dataset.

Table S1. Description of the ISO 3-alpha code (part 1).

ISO 3-alpha code	Country name	ISO 3-alpha code	Country name
ABW	Aruba	DNK	Denmark
AFG	Afghanistan	DOM	Dominican Republic
AGO	Angola	DZA	Algeria
ALB	Albania	ECU	Ecuador
AND	Andorra	EGY	Egypt
ARE	United Arab Emirates	ERI	Eritrea
ARG	Argentina	ESP	Spain
ARM	Armenia	EST	Estonia
ATG	Antigua and Barbuda	ETH	Ethiopia
AUS	Australia	FIN	Finland
AUT	Austria	FJI	Fiji
AZE	Azerbaijan	FLK	Falkland Islands (Malvinas)
BDI	Burundi	FRA	France
BEL	Belgium-Luxembourg	FSM	Micronesia (Federated States of)
BEN	Benin	GAB	Gabon
BFA	Burkina Faso	GBR	United Kingdom
BGD	Bangladesh	GEO	Georgia
BGR	Bulgaria	GHA	Ghana
BHR	Bahrain	GIB	Gibraltar
BHS	Bahamas	GIN	Guinea
BIH	Bosnia and Herzegovina	GMB	Gambia
BLR	Belarus	GNB	Guinea-Bissau
BLZ	Belize	GNQ	Equatorial Guinea
BMU	Bermuda	GRC	Greece
BOL	Bolivia	GRD	Grenada
BRA	Brazil	GRL	Greenland
BRB	Barbados	GTM	Guatemala
BRN	Brunei Darussalam	GUM	Guam
BTN	Bhutan	GUY	Guyana
CAF	Central African Republic	HKG	Hong Kong (SARC)
CAN	Canada	HND	Honduras
CHE	Switzerland-Liechtenstein	HRV	Croatia
CHL	Chile	HTI	Haiti
CHN	China	HUN	Hungary
CIV	Côte d'Ivoire	IDN	Indonesia
CMR	Cameroon	IND	India
COD	Democratic Republic of the Congo	IRL	Ireland
COG	Congo	IRN	Iran (Islamic Republic of)
COL	Colombia	IRQ	Iraq
COM	Comoros	ISL	Iceland
CPV	Cape Verde	ISR	Israel
CRI	Costa Rica	ITA	Italy
CUB	Cuba	JAM	Jamaica
CYM	Cayman Islands	JOR	Jordan
CYP	Cyprus	JPN	Japan
CZE	Czech Republic	KAZ	Kazakstan
DEU	Germany	KEN	Kenya
DJI	Djibouti	KGZ	Kyrgyzstan
DMA	Dominica	KHM	Cambodia

Table S2. Description of the ISO 3-alpha code (part 2).

ISO 3-alpha code	Country name	ISO 3-alpha code	Country name
KNA	Saint Kitts and Nevis	PYF	French Polynesia
KOR	Korea, Rep. of Korea	QAT	Qatar
KWT	Kuwait	ROU	Roumania
LAO	Lao People's Democratic Republic	RUS	Russian Federation
LBN	Lebanon	RWA	Rwanda
LBR	Liberia	SAU	Saudi Arabia
LBY	Libyan Arab Jamahiriya	SDN	Sudan
LCA	Saint Lucia	SEN	Senegal
LKA	Sri Lanka	SGP	Singapore
LTU	Lithuania	SLB	Solomon Islands
LVA	Latvia	SLE	Sierra Leone
MAC	Macau	SLV	El Salvador
MAR	Morocco	SMR	San Marino
MDA	Moldova, Rep.of	SOM	Somalia
MDG	Madagascar	SUR	Suriname
MDV	Maldives	SVK	Slovakia
MEX	Mexico	SVN	Slovenia
MHL	Marshall Islands	SWE	Sweden
MKD	The former Yugoslav Rep. of Macedonia	SYC	Seychelles
MLI	Mali	SYR	Syrian Arab Republic
MLT	Malta	TCD	Chad
MMR	Myanmar	TGO	Togo
MNG	Mongolia	THA	Thailand
MOZ	Mozambique	TJK	Tajikistan
MRT	Mauritania	TKM	Turkmenistan
MUS	Mauritius	TLS	East Timor
MWI	Malawi	TTO	Trinidad and Tobago
MYS	Malaysia	TUN	Tunisia
NCL	New Caledonia	TUR	Turkey
NER	Niger	TUV	Tuvalu
NFK	Norfolk Island	TZA	Tanzania, United Rep. of
NGA	Nigeria	UGA	Uganda
NIC	Nicaragua	UKR	Ukraine
NIU	Niue	URY	Uruguay
NLD	Netherlands	USA	United States of America
NOR	Norway	UZB	Uzbekistan
NPL	Nepal	VCT	Saint Vincent and the Grenadines
NZL	New Zealand	VEN	Venezuela
OMN	Oman	VGB	British Virgin Islands
PAK	Pakistan	VNM	Viet Nam
PAN	Panama	VUT	Vanuatu
PER	Peru	WSM	Samoa
PHL	Philippines	YEM	Yemen
PNG	Papua New Guinea	ZAF	South Africa
POL	Poland	ZMB	Zambia
PRK	Korea, Dem. People's Rep. of	ZWE	Zimbabwe
PRT	Portugal		
PRY	Paraguay		
PSE	State of Palestine		

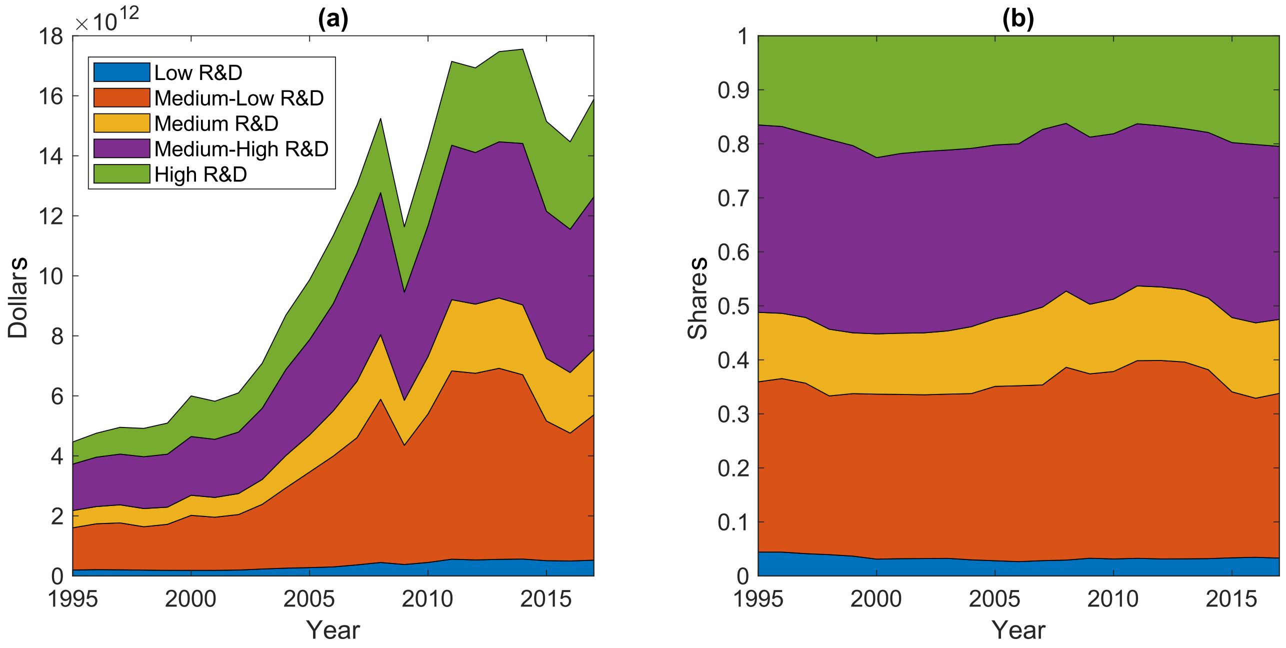

Figure S2. Time series of the traded dollars grouped by the R&D classes. Panel (a) shows the traded dollars, while panel (b) reports the market shares in each R&D class. The Figure is produced with MATLAB R2020b.

Author contributions statement

L.C., F.L, L.R., and C.S. conceived and designed the study. L.C. performed the experiments. L.C., F.L, L.R., and C.S. analysed the results. L.C. wrote the manuscript and made all the figures. F.L, L.R., and C.S. edited the manuscript. All authors reviewed the manuscript.

Additional information

To include, in this order: **Accession codes** (where applicable); **Competing interests** (mandatory statement).

The corresponding author is responsible for submitting a competing interests statement on behalf of all authors of the paper. This statement must be included in the submitted article file.

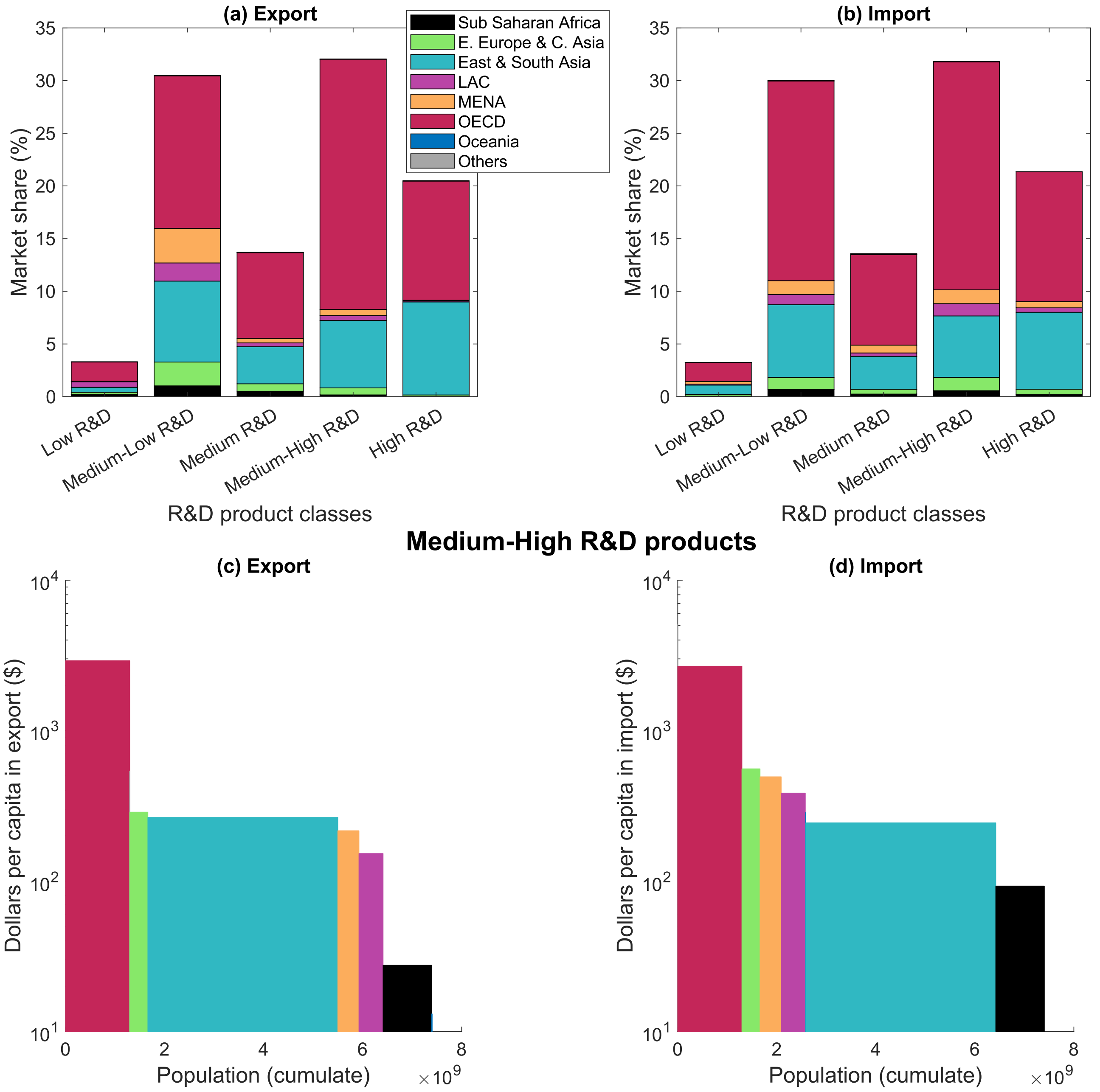

Figure S3. Description of the international trade in terms of products' R&D intensity and world regions defined according to Sachs et al.[1]. The top panels show the shares of global trade held by regions in each class of R&D intensity both in export (panel (a)) and import (panel (b)) in 2017 (thus, showing the elements of $\mathbf{MR_{exp}}$ and $\mathbf{MR_{imp}}$, please see Equation (S1) of this document). The bottom panels report the per-capita dollars traded by all regions (sorted from the largest to the smallest) for the products in the Medium-High R&D class in 2017 as a function of the population. The Figure is produced with MATLAB R2020b.

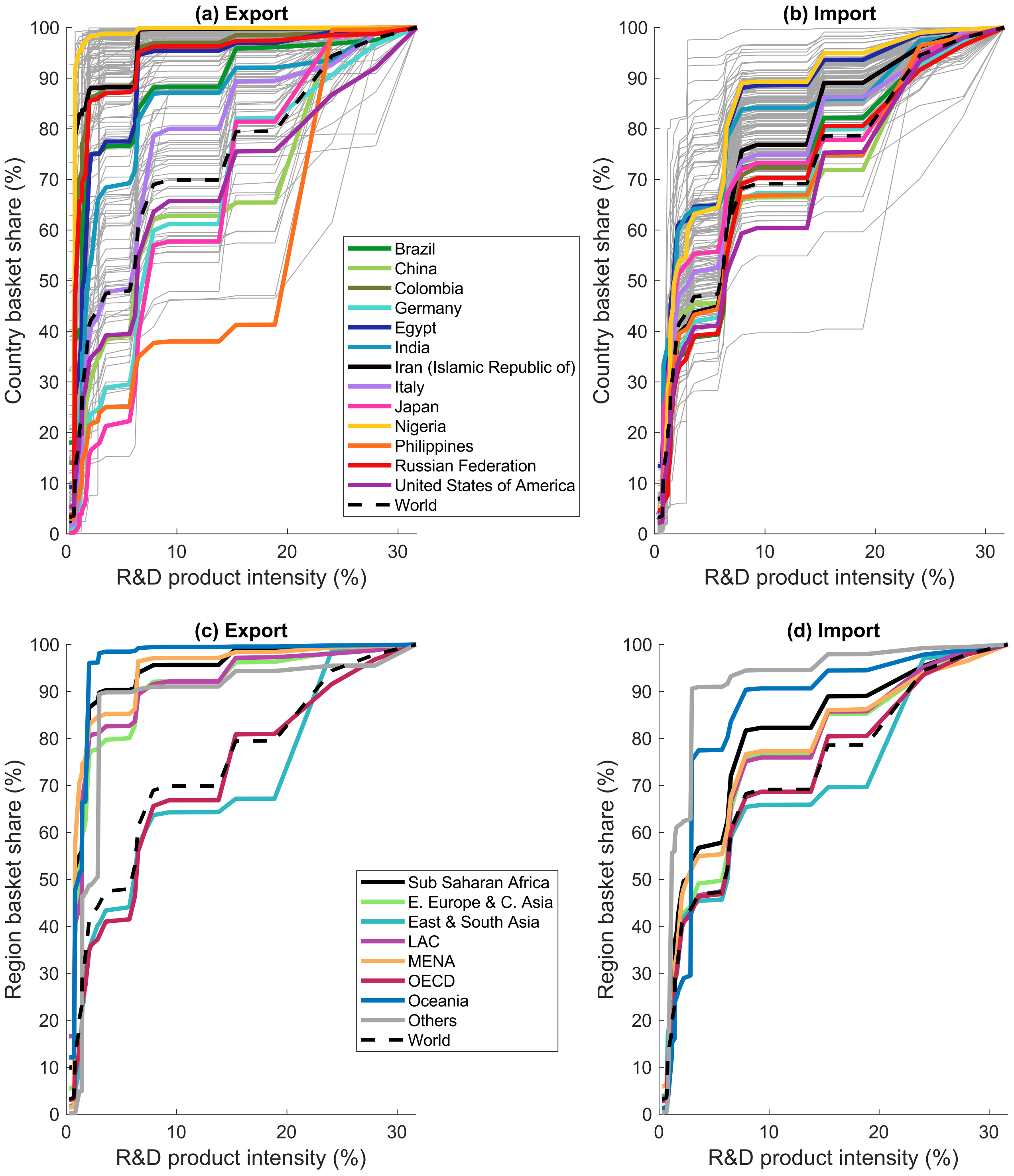

Figure S4. Export and import basket shares for countries (panels (a) and (b), respectively) and regions (panels (c) and (d)) as a function of the R&D intensity of the traded goods in the year 2017. The Figure is produced with MATLAB R2020b.

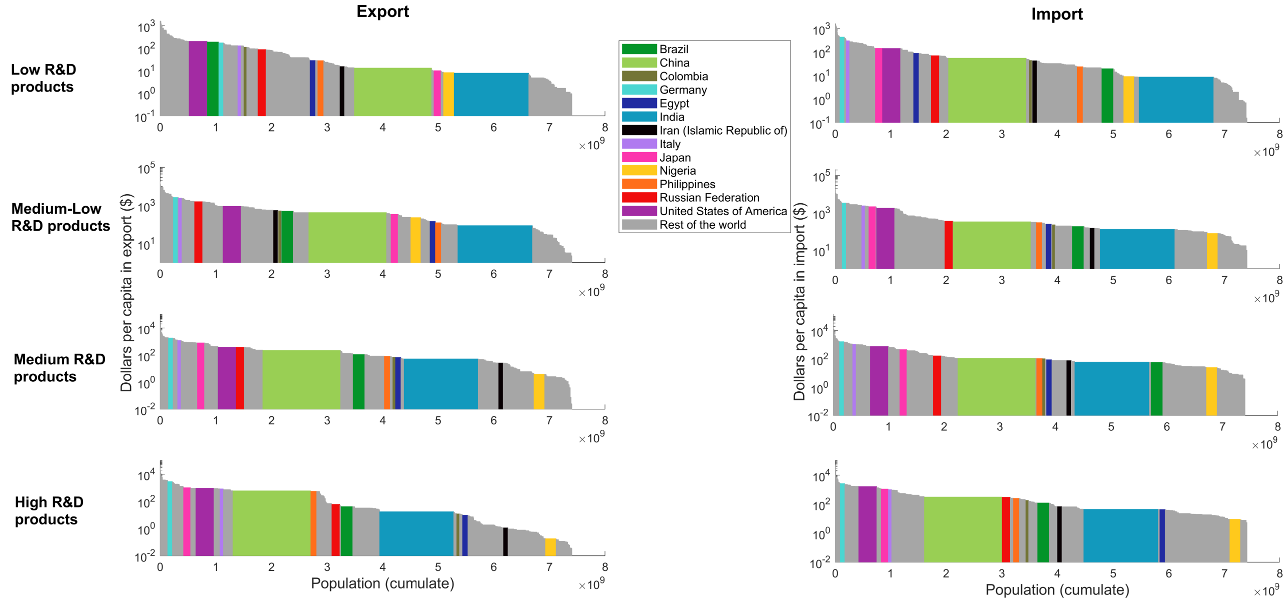

Figure S5. Countries' per-capita dollars in export (left panels) and import (right panels) in the R&D classes not shown in Figure 2 of the main text. Countries are sorted from the highest per-capita dollars exported and imported to the lowest. All panels refer to the year 2017. The Figure is produced with MATLAB R2020b.

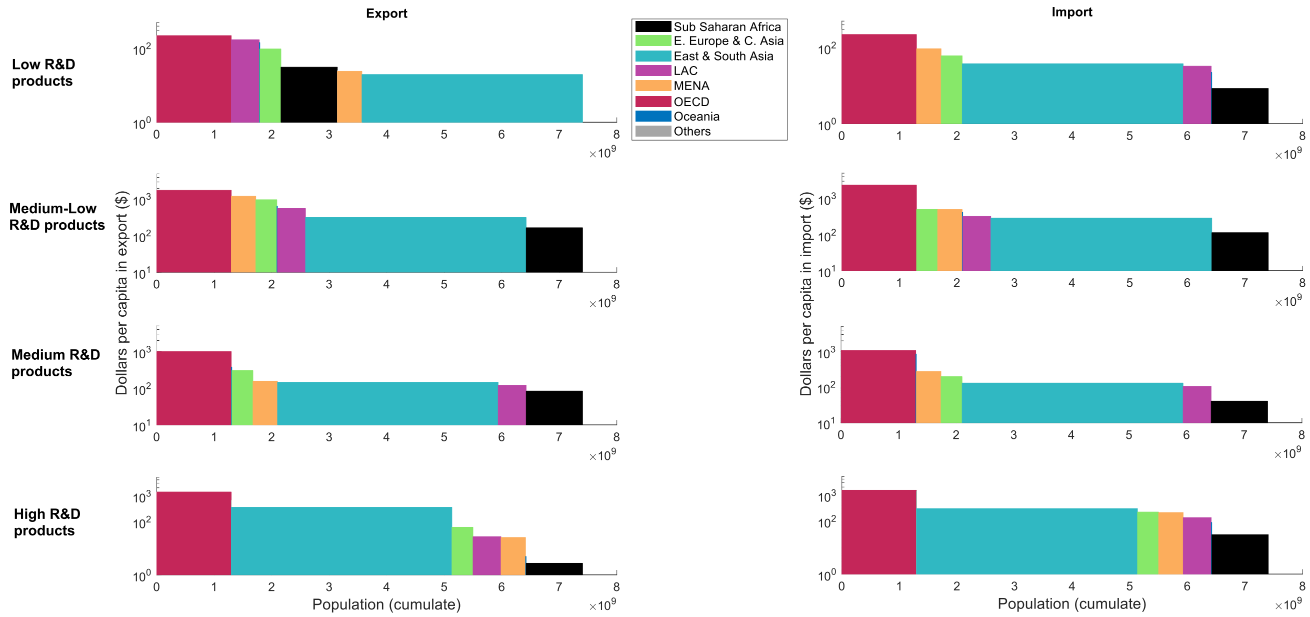

Figure S6. Regions' per-capita dollars in export (left panels) and import (right panels) in the R&D classes not shown in Figure S2 of this SM. Countries are sorted from the highest per-capita dollars exported and imported to the lowest. All panels refer to the year 2017. The Figure is produced with MATLAB R2020b.

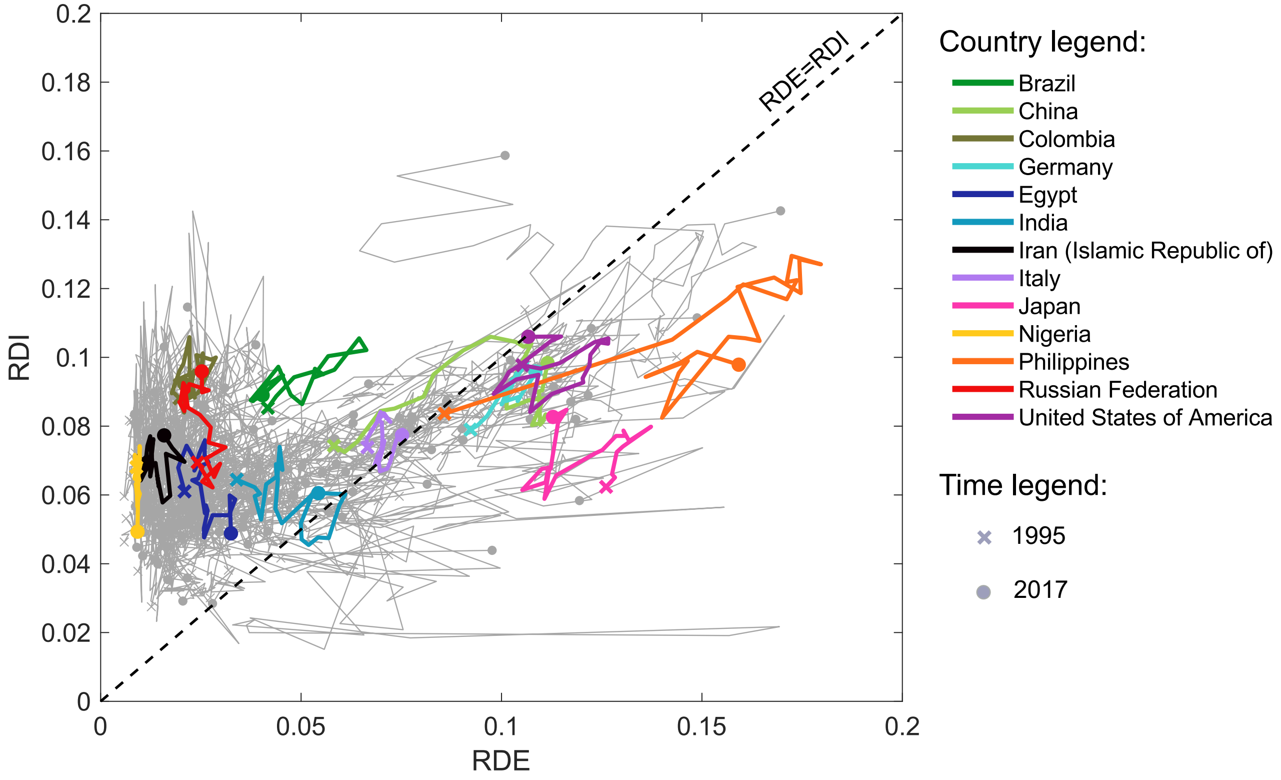

Figure S7. Countries' trajectories in the RDI-RDE plane between 1995 and 2017, highlighting those of Brazil, China, Colombia, Germany, Egypt, India, Iran, Italy, Japan, Nigeria, the Philippines, the Russian Federation, and the United States of America, as referred in the main text. The black dashed line reports when the RDI and RDE are equal. The Figure is produced with MATLAB R2020b.

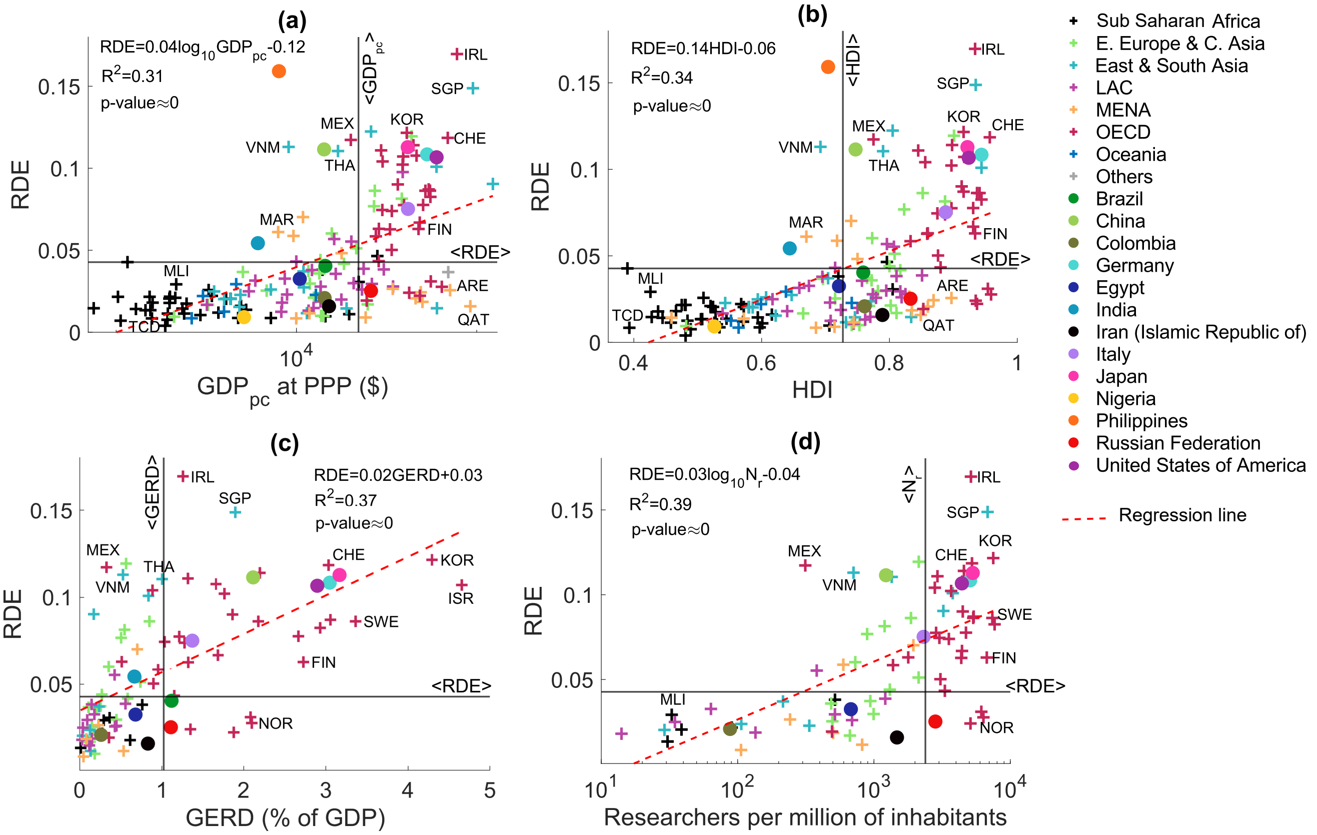

Figure S8. Comparison between countries' RDE values and their socio-economic characteristics. Here, the ISO 3-alpha code is used to tag countries, and correspondence is given in Tables S1 and S2. Panel (a) compares the RDE with the per capita Gross Domestic Product at Purchasing Power Parity in constant 2017 dollars (GDP_{pc} at PPP), panel (b) with the Human Development Index (HDI), panel (c) with the Gross Expenditure in Research and Development as a percentage of GDP (GERD), and panel (d) for the number of researchers per million of inhabitants (N_r) for the year 2017. The colour of the plus signs refers to the organisation/geographical region the country belongs to as defined in Sachs et al.[1]. Filled dots identify the 13 countries highlighted as examples throughout the text. Panels (a)-(d) are accompanied by the statistical description of the regression lines (dashed red lines). Here, the thick black lines mark the average values of the variables. The Figure is produced with MATLAB R2020b.

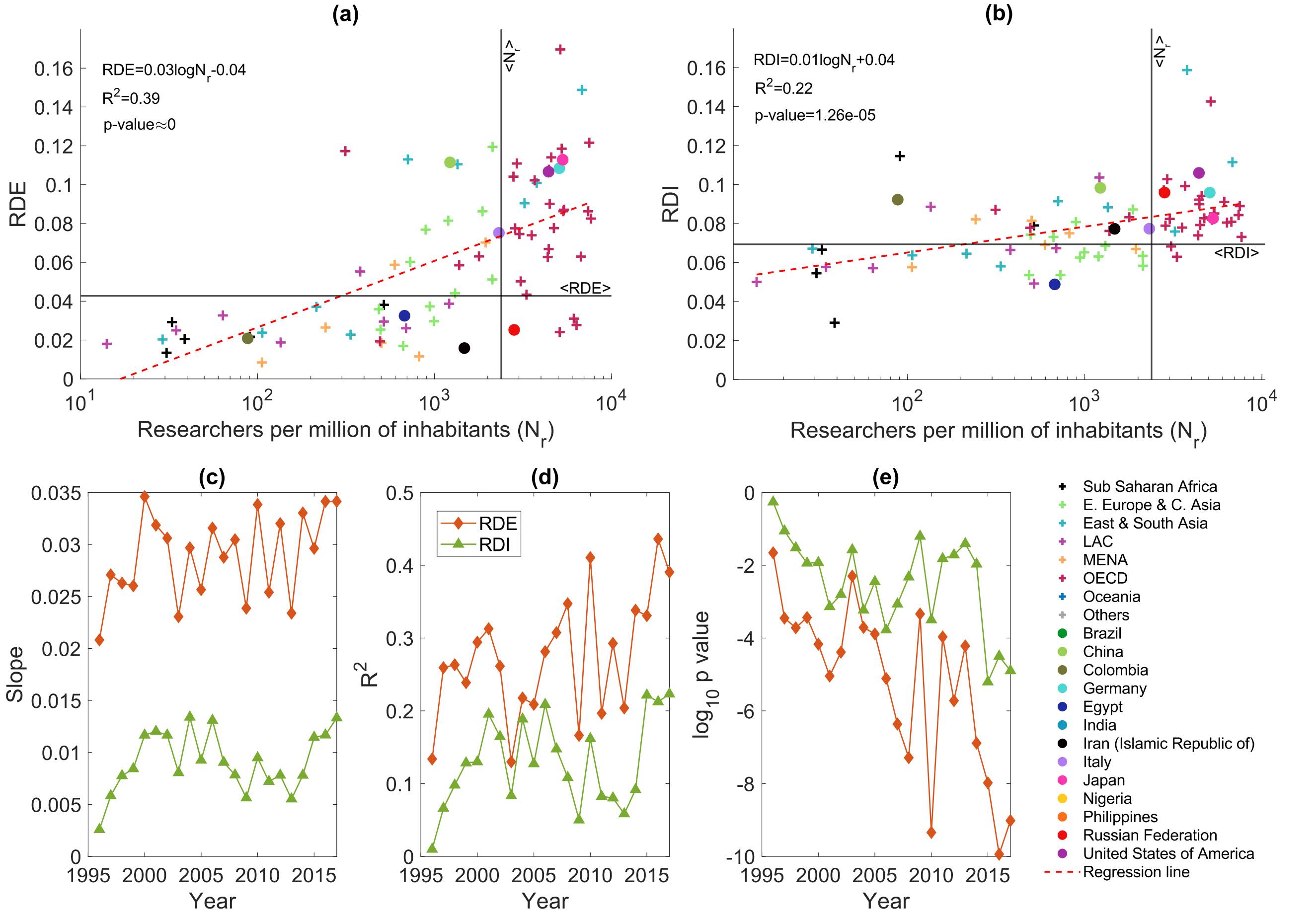

Figure S9. Comparison between the RDE – panel (a) – (RDI – panel (b)) and the number of researchers per million of inhabitants (N_r). The colour of the plus signs refers to the organisation/geographical region the country belongs to according to Sachs et al.[1]. Filled dots identify the 13 countries highlighted as examples throughout the text. Top panels refer to the year 2017. The fit was developed for all of the years in the period 1995 - 2017. Thus, the bottom panels (panels (c), (d), and (e)) report the time series of the fit parameters. The Figure is produced with MATLAB R2020b.

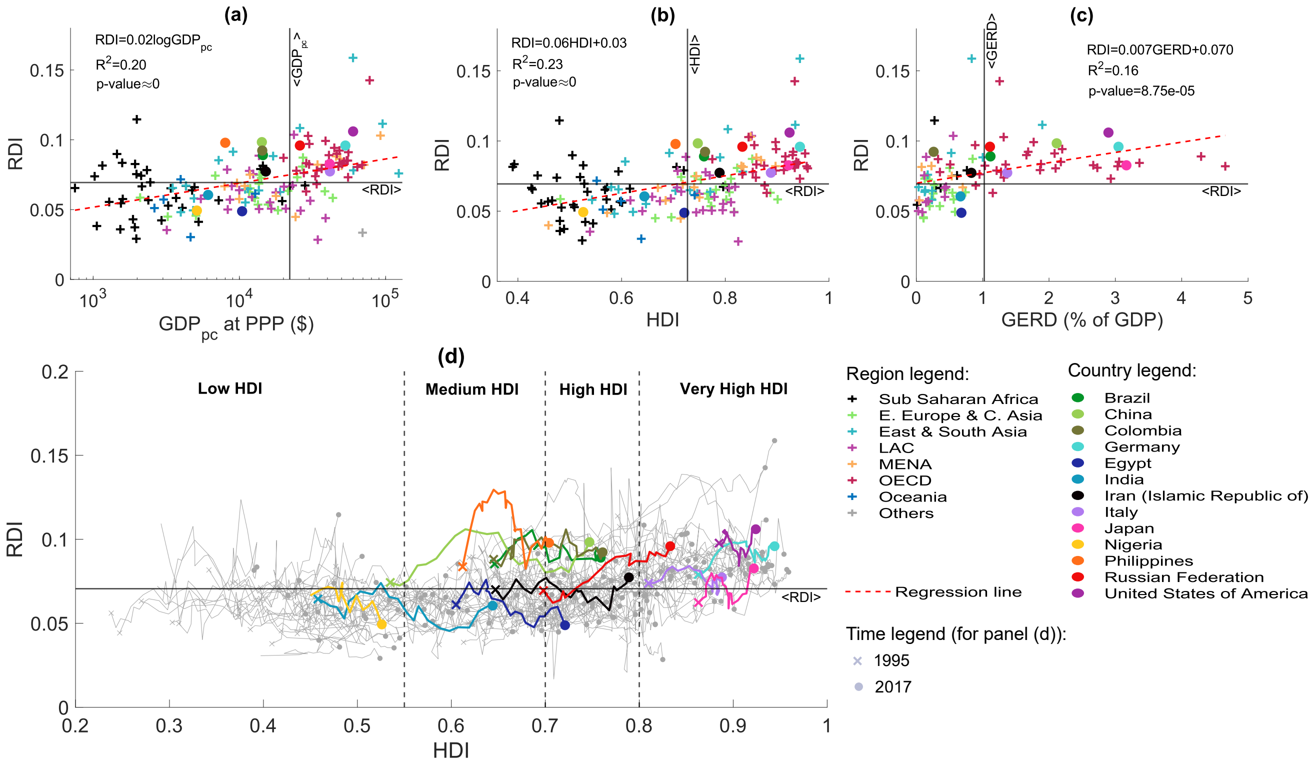

Figure S10. Comparison between countries' RDI values and their Gross Domestic Product per capita at Purchasing Power Parity in constant 2017 dollars (GDP_{pc} at PPP, panel (a)), Human Development Index (HDI, panel (b)), and Gross Expenditure in Research and Development as a percentage of GDP (GERD, panel (c)) for the year 2017. The colour of the plus signs refers to the organisation/geographical region the country belongs as defined in Sachs et al.[1]. Filled dots identify the 13 countries highlighted as examples throughout the text. Panels (a)-(c) are accompanied by the statistical description of the regression lines (dashed red lines). Here, the thick black lines mark the average values of the variables. Panel (d) shows the trajectories in the RDI-HDI plane for all countries in analysis (grey thin lines), highlighting those of Brazil, China, Colombia, Germany, Egypt, India, Iran, Italy, Japan, Nigeria, the Philippines, the Russian Federation, and the United States of America, as referred in the main text (coloured lines). In this panel, the dashed black lines bound classes of HDI values as defined by the United Nations[2]. The Figure is produced with MATLAB R2020b.

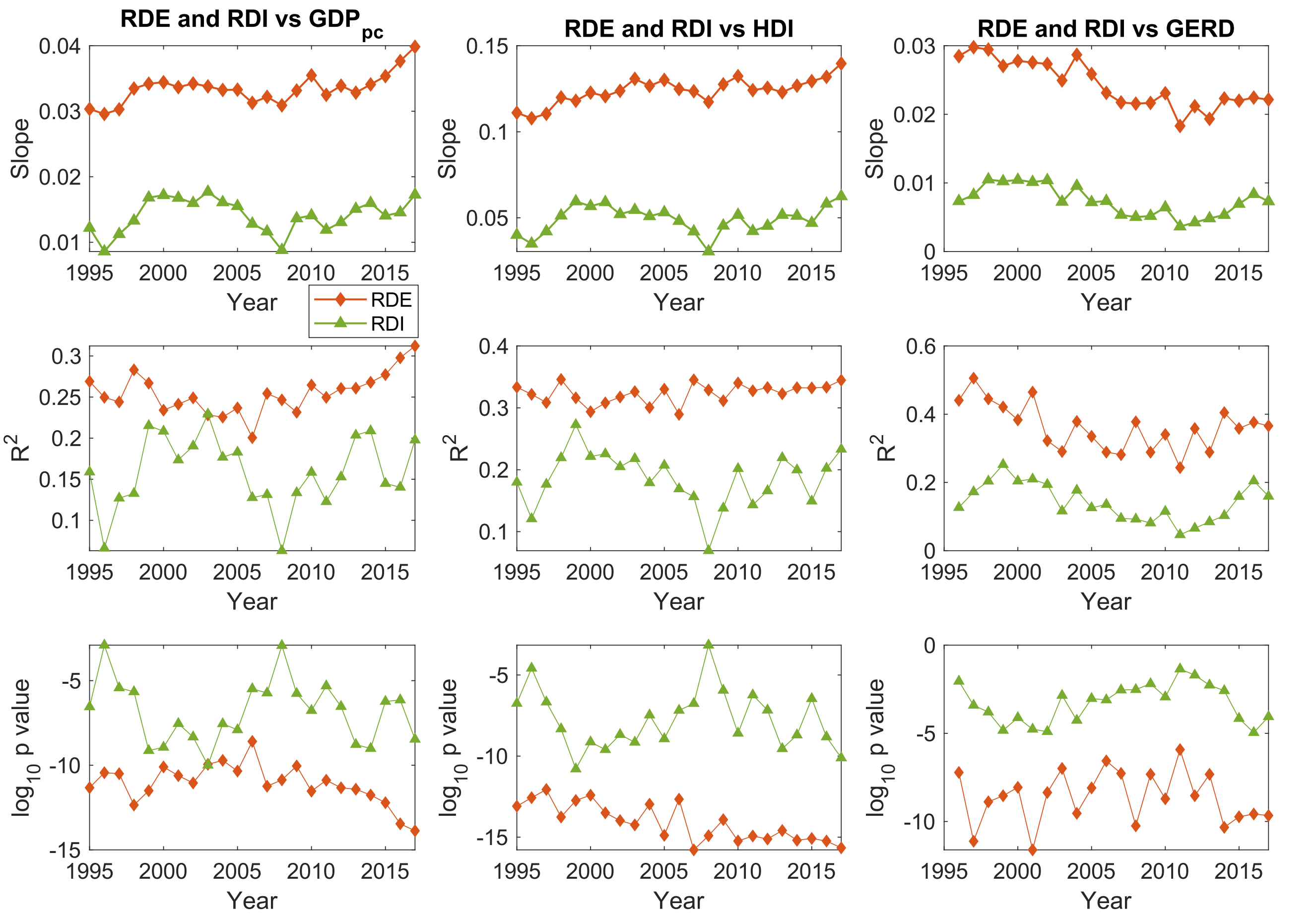

Figure S11. Parameters of the fits between the RDE (red diamonds) and RDI (green triangles) values vs the GDP_{pc} (left column), HDI (central column), and GERD values (right column). In vertical reading, the fit parameters are slope (top row), R^2 (middle row), and p-value (bottom row). The Figure is produced with MATLAB R2020b.

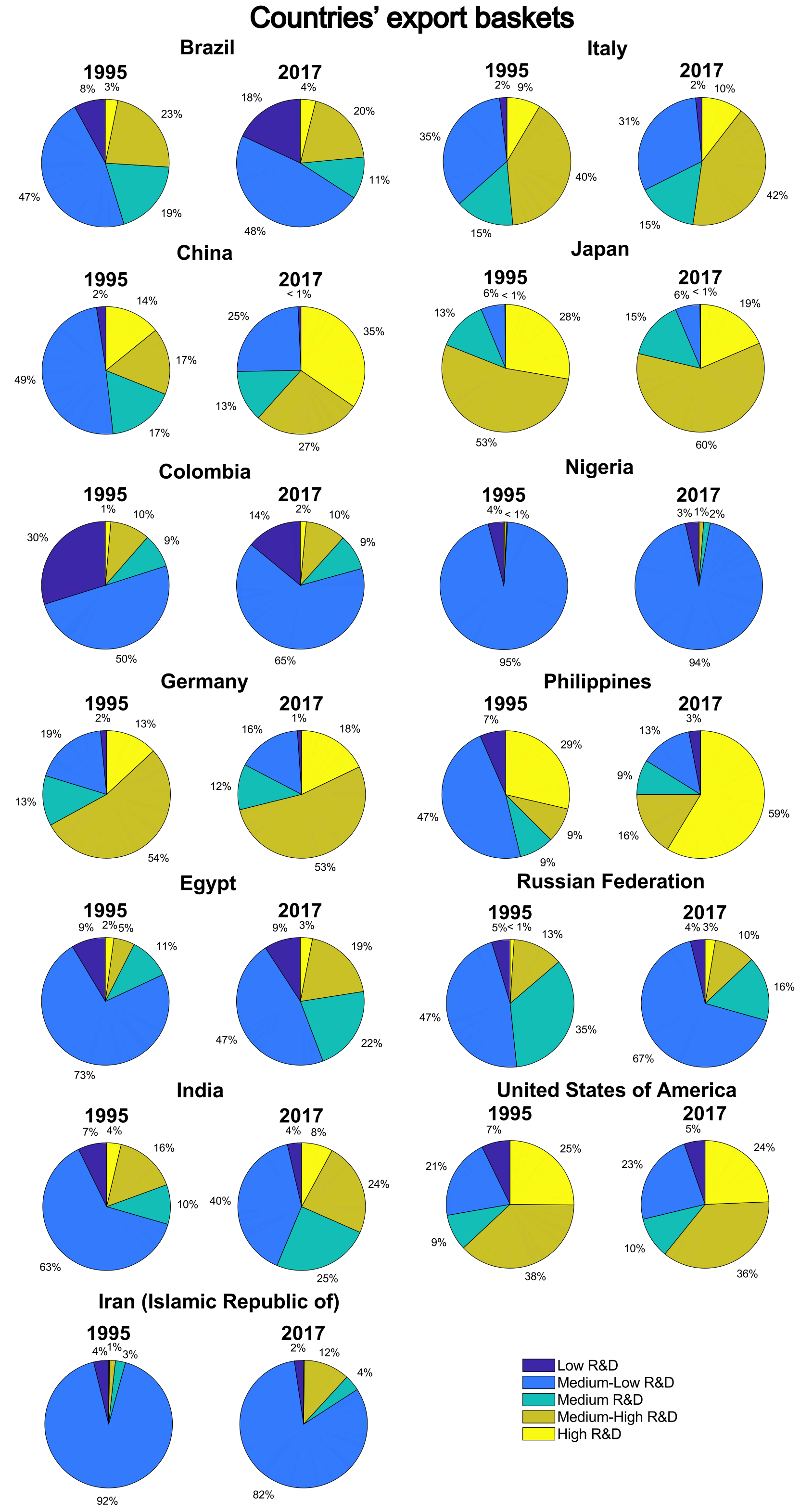

Figure S12. Description of the export baskets of the thirteen countries highlighted in the main text in terms of R&D classes in 1995 and 2017. The Figure is produced with MATLAB R2020b.

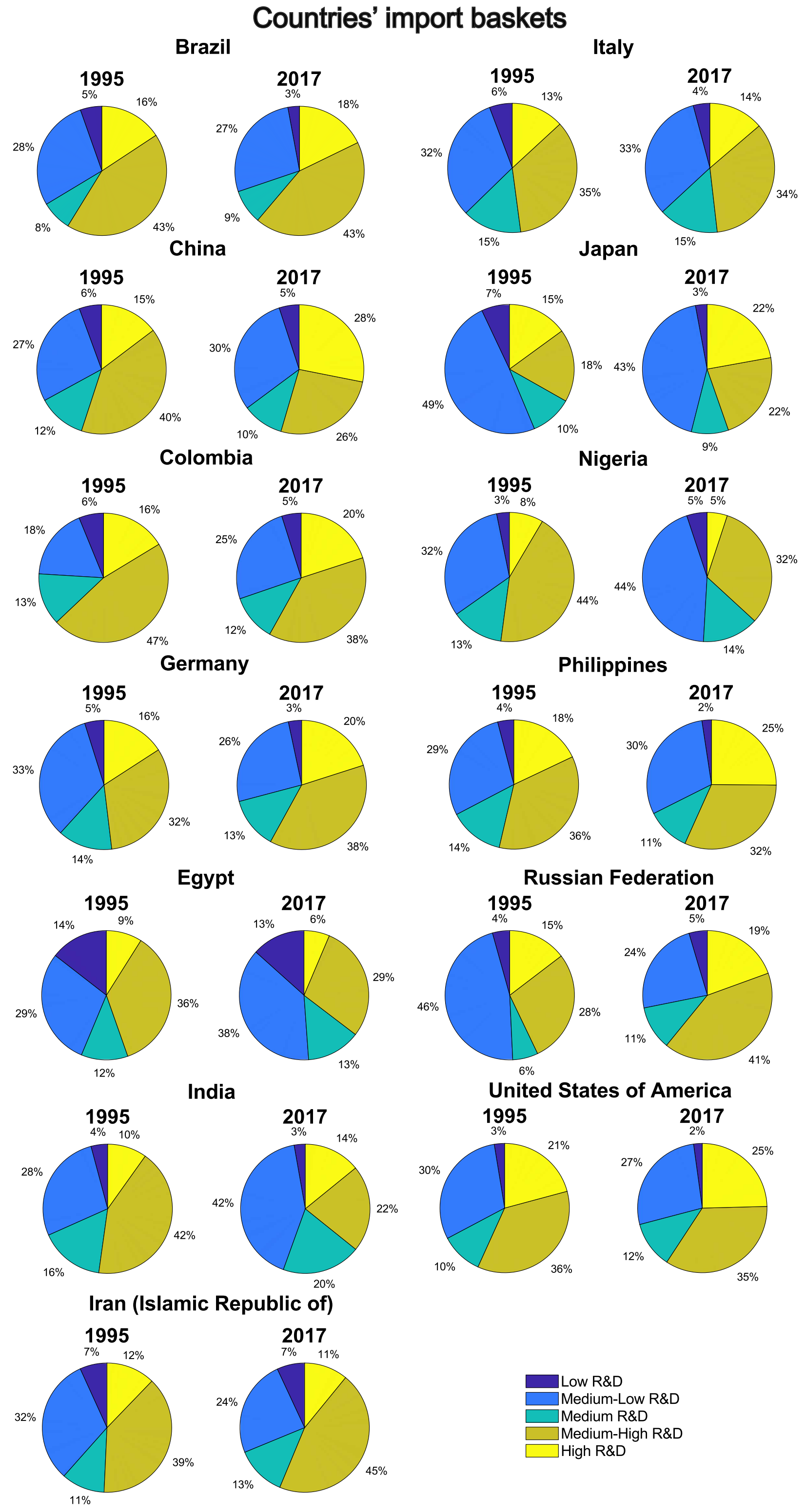

Figure S13. Description of the import baskets of the thirteen countries highlighted in the main text in terms of R&D classes in 1995 and 2017. The Figure is produced with MATLAB R2020b.

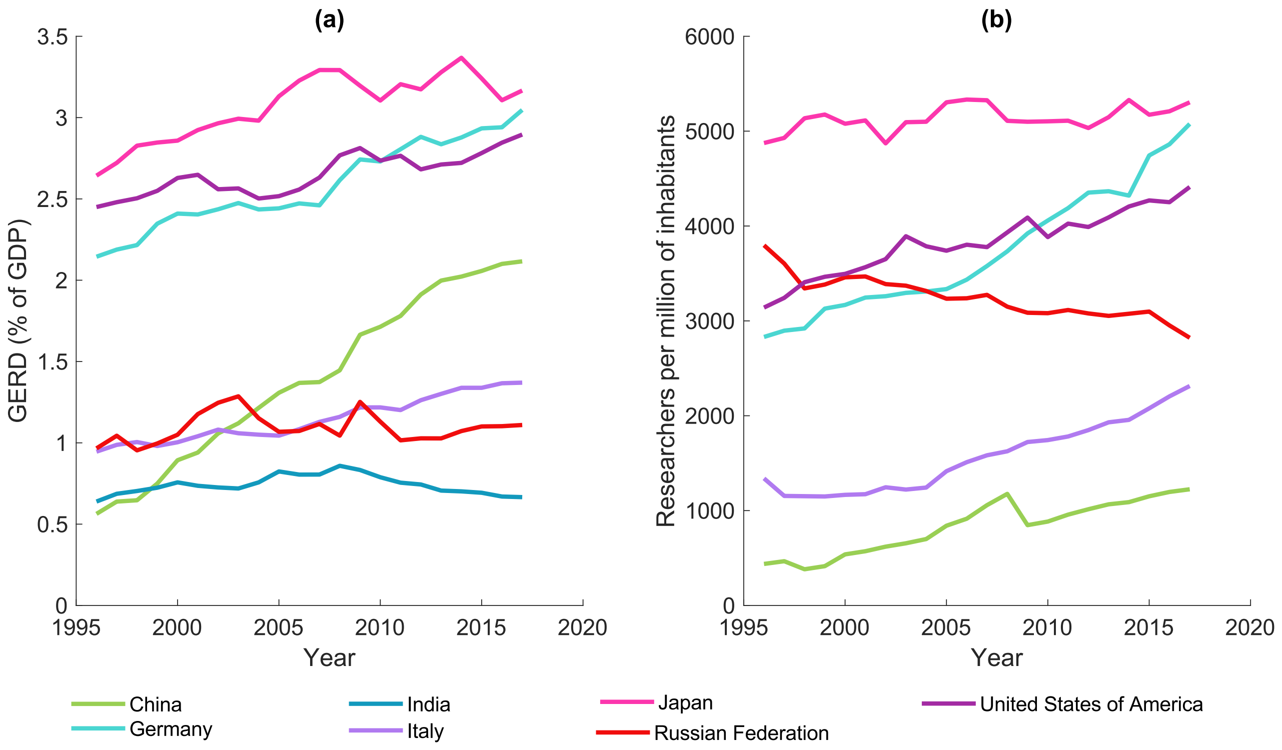

Figure S14. Time series of the Gross Expenditure on R&D (GERD, panel (a)) and number of researchers per million of inhabitants (panel (b)) for the highlighted countries. Please, note that the figure shows only countries with full records between 1995 and 2017. The Figure is produced with MATLAB R2020b.

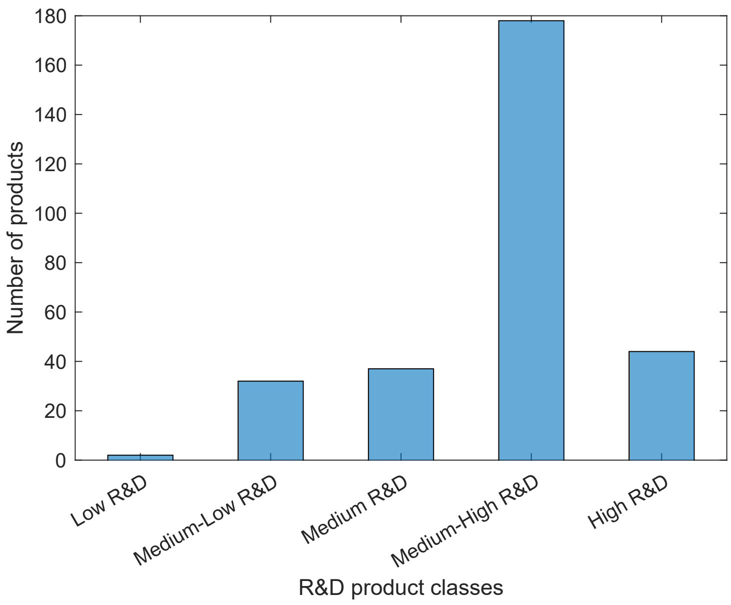

Figure S15. Number of green products for each R&D class. The Figure is produced with MATLAB R2020b.

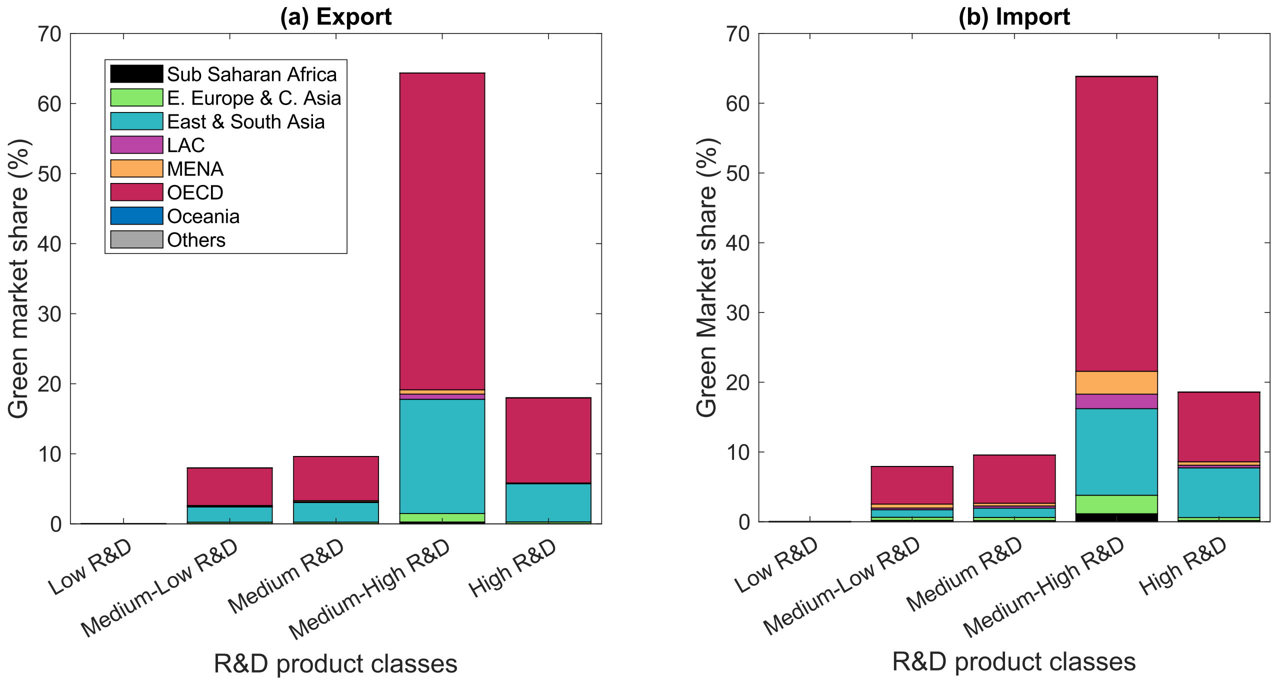

Figure S16. Regions' shares of green market in each R&D class both in export (panel (a)) and import (panel (b)) in 2017 (computed applying Equation (S2) of this document). The Figure is produced with MATLAB R2020b.

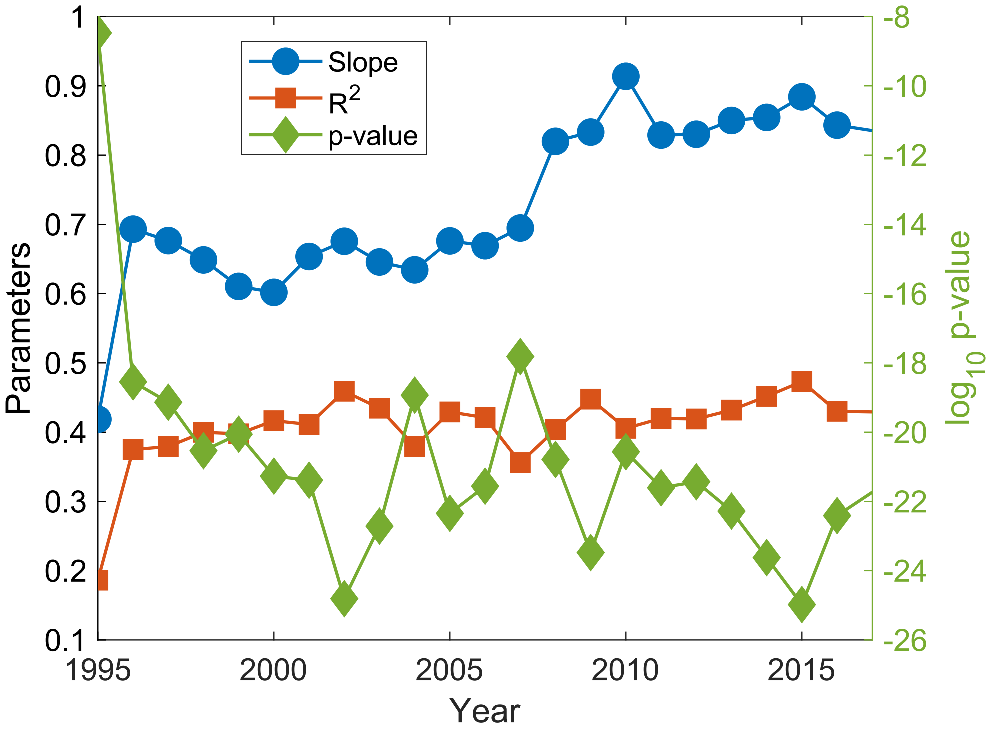

Figure S17. Linear fit parameters (slope, R^2, and p-value) between countries' greenness and RDE in time. The Figure is produced with MATLAB R2020b.

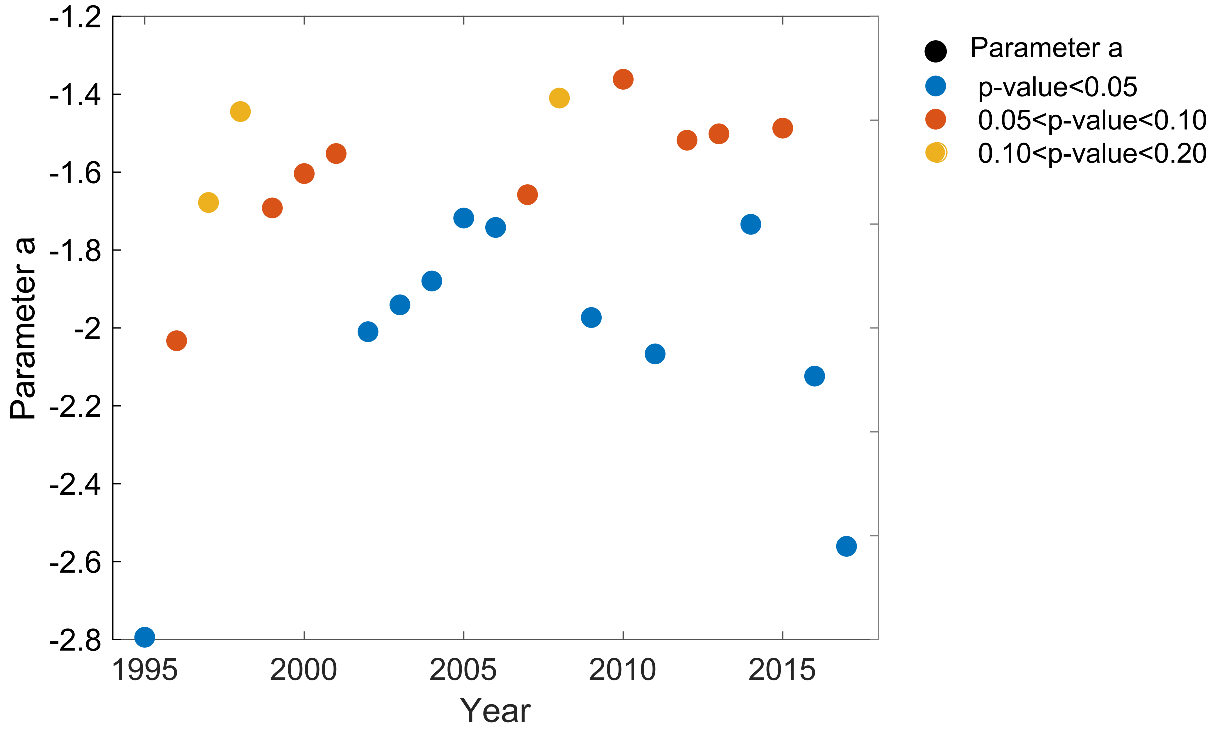

Figure S18. Parameter *a* of the fit between countries' CO_2 export intensity and RDE values in time. The colour of the dots reports the associated p-value ranges. The Figure is produced with MATLAB R2020b.

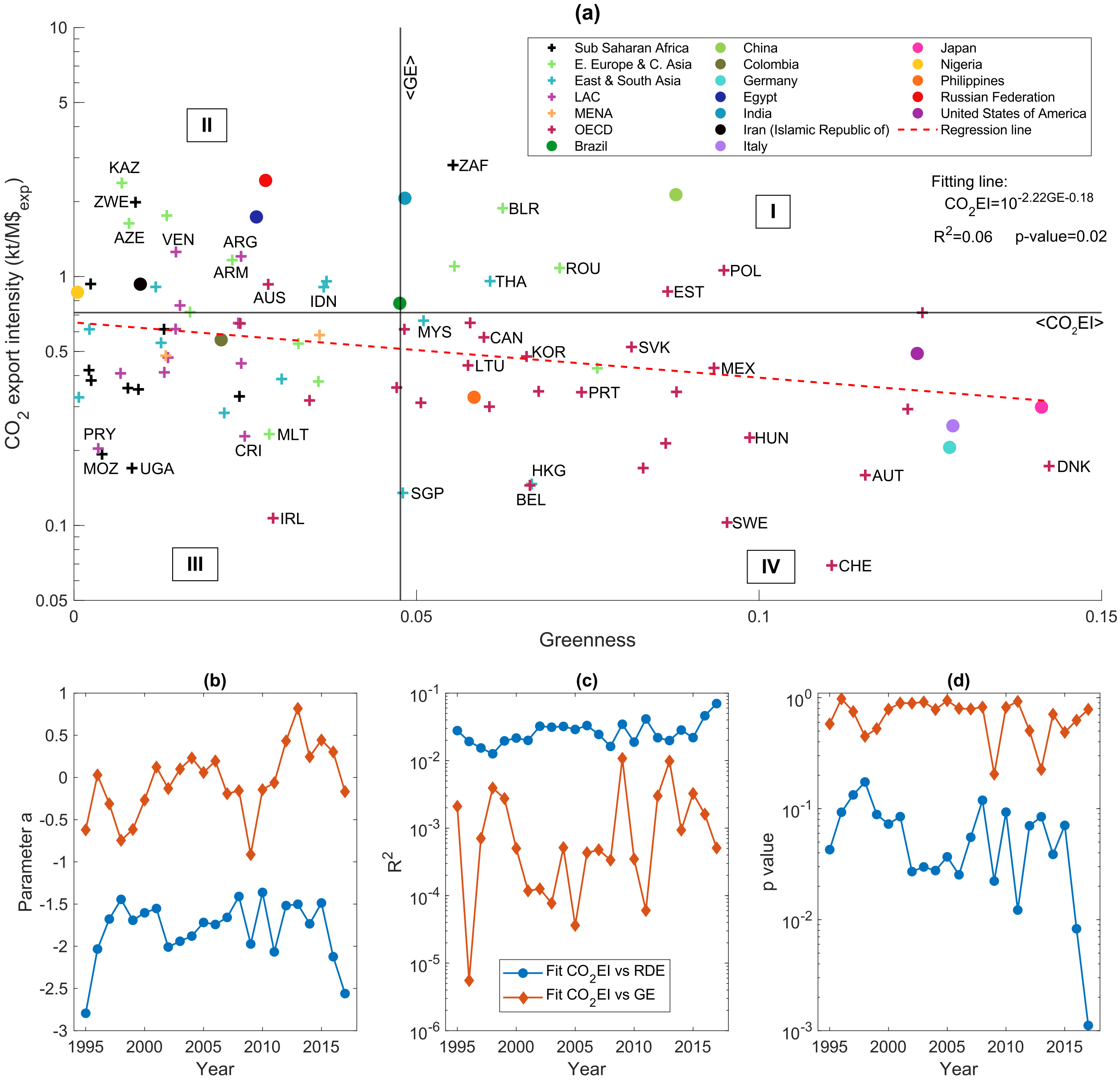

Figure S19. Comparison between CO_2 export intensity (CO_2EI) and the countrie's greenness (GE). Panel (a) shows this comparison using CO_2 data from Davis et al.[6], and it thus refer to the year 2004. Here, the ISO 3-alpha code is used to tag countries, and correspondence is given in Tables S1 and S2. The colour of the plus signs refers to the organisation/geographical region the country belongs to according to Sachs et al.[1]. Filled dots highlight the 13 countries highlighted throughout the main text. In panel (a), the red line represents the fitting line, and its equation and statistical meaning are thereby detailed. The fit has been developed for all of the years during the period 1995 - 2017, using the CO_2 values estimated using the data from the Global Carbon Budget[4] and the World Bank[5]. Thus, the bottom panels (panels (b), (c), and (d)) report the fit parameter in time and also provide information about the fit parameters obtained by comparing the CO_2 export intensity and the RDE values. The Figure is produced with MATLAB R2020b.

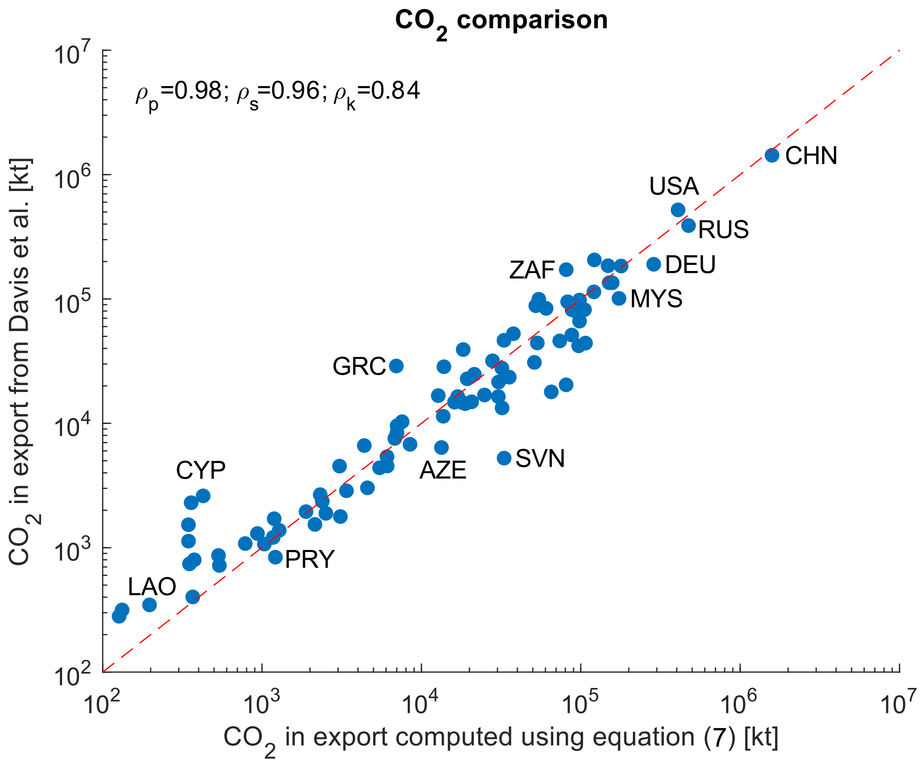

Figure S20. Comparison between the CO_2 embedded in the export estimated using Equation (7) of the main text and that published by Davis et al.[6]. The scatter refers to the year 2004, the only year available in the work of Davis et al.[6]. Here, the ISO 3-alpha code is used to tag countries, and correspondence is given in Tables S1 and S2. The dashed red line indicates the equality between the quantities along the axes. The text-box reports the correlation of Pearson (ρ_p), Spearman (ρ_s), and Kendall (ρ_k). The Figure is produced with MATLAB R2020b.

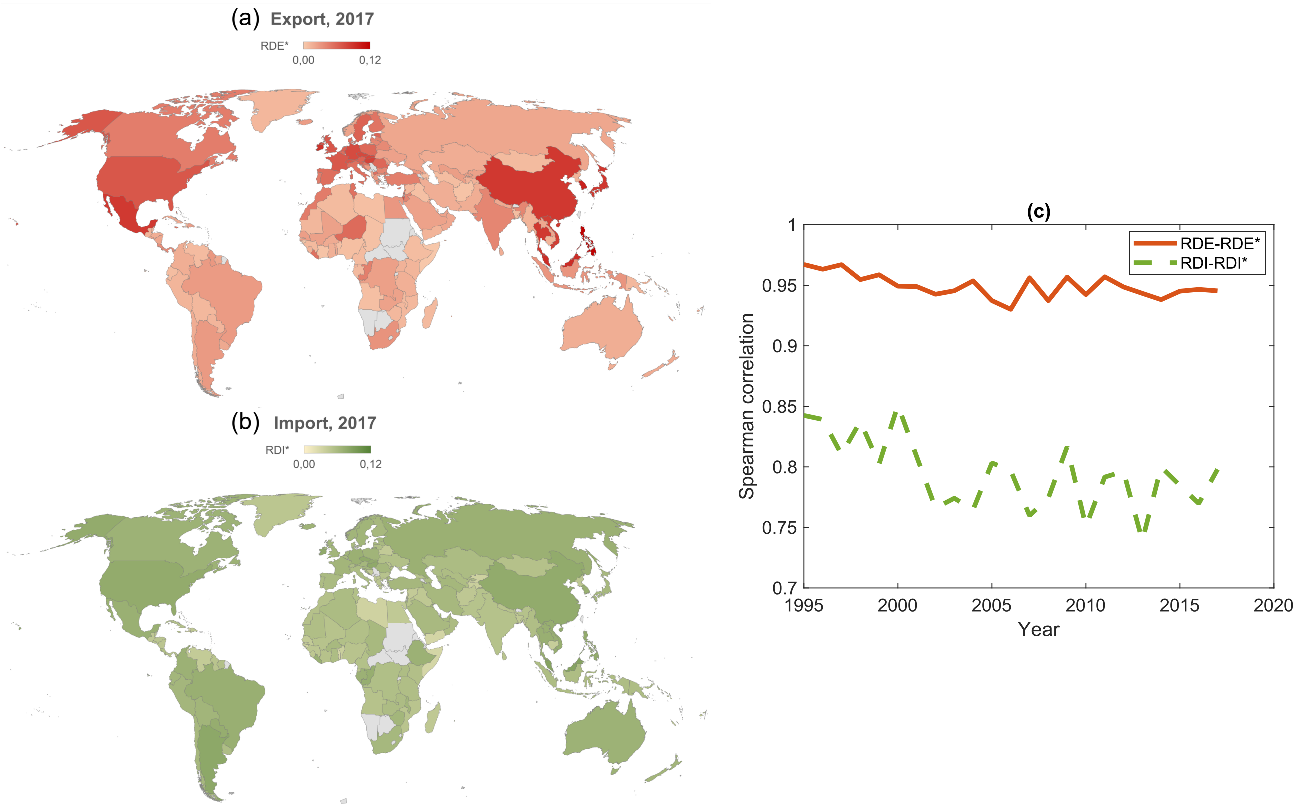

Figure S21. Research and Development embedded in the export (RDE*) and import((RDI*) baskets of countries worldwide computed accounting for China's R&D data in accordance with [7] and as detailed in this SM (see, Eqs. (3)-(4) in the main text). The left panels show the geographical distribution of these indices for 2017 for the export (panel (a)) and import (panel (b)) baskets, respectively, considering the R&D intensity value for each industry. Panel (c) shows the Spearman's correlation values in time computed among the values RDE-RDE* and RDI-RDI*, i.e., how correlated the values of the indices are when China's data are included in the definition of the R&D intensities. The Figure is produced with Excel 2019 and MATLAB R2020b.
